# Increased Power for Detection of Parent-of-Origin Effects via the Use of Haplotype Estimation

**DOI:** 10.1016/j.ajhg.2015.07.016

**Published:** 2015-08-27

**Authors:** Richard Howey, Chrysovalanto Mamasoula, Ana Töpf, Ron Nudel, Judith A. Goodship, Bernard D. Keavney, Heather J. Cordell

**Affiliations:** 1 Institute of Genetic Medicine, Newcastle University, Newcastle upon Tyne, NE1 3BZ, UK; 2 Institute of Health and Society, Newcastle University, Newcastle upon Tyne, NE2 4AX, UK; 3 Wellcome Trust Centre for Human Genetics, University of Oxford, Oxford, OX3 7BN, UK; 4 Institute of Cardiovascular Sciences, University of Manchester, Manchester, M13 9NT, UK

## Abstract

Parent-of-origin (or imprinting) effects relate to the situation in which traits are influenced by the allele inherited from only one parent and the allele from the other parent has little or no effect. Given SNP genotype data from case-parent trios, the parent of origin of each allele in the offspring can often be deduced unambiguously; however, this is not true when all three individuals are heterozygous. Most existing methods for investigating parent-of-origin effects operate on a SNP-by-SNP basis and either perform some sort of averaging over the possible parental transmissions or else discard ambiguous trios. If the correct parent of origin at a SNP could be determined, this would provide extra information and increase the power for detecting the effects of imprinting. We propose making use of the surrounding SNP information, via haplotype estimation, to improve estimation of parent of origin at a test SNP for case-parent trios, case-mother duos, and case-father duos. This extra information is then used in a multinomial modeling approach for estimating parent-of-origin effects at the test SNP. We show through computer simulations that our approach has increased power over previous approaches, particularly when the data consist only of duos. We apply our method to two real datasets and find a decrease in significance of p values in genomic regions previously thought to possibly harbor imprinting effects, thus weakening the evidence that such effects actually exist in these regions, although some regions retain evidence of significant effects.

## Introduction

Parent-of-origin effects relate to the situation where traits are influenced by the allele inherited from only one parent (e.g., the mother), with the allele from the other parent (e.g., the father) having little or no effect. More generally, parent-of-origin effects can be defined as effects where the alleles inherited from the different parents have differing effects on some phenotype of interest. This phenomenon is not the same as a direct effect of maternal genotype. A maternal-genotype effect occurs when an offspring’s phenotype is altered (perhaps in utero) by the maternal genotype, regardless of the allele actually transmitted to the child. One biological mechanism that can lead to parent-of-origin effects is genomic imprinting, the phenomenon whereby either the maternally or the paternally inherited allele is expressed, while the other allele is silenced. The mechanisms underlying imprinting are not yet fully understood, but are believed to involve epigenetic processes including histone acetylation and DNA methylation.^1^

Parent-of-origin effects due to genomic imprinting (or due to interactions with imprinted loci) have been observed for multiple traits in outbred mice.^2^ In humans, parent-of-origin effects have been observed at known imprinted regions for a variety of phenotypes,^3^ including chromosome 14q32 in type 1 diabetes,^4^ chromosome 7q32 in type 2 diabetes,^5^ and chromosome 11p15 in breast cancer.^5^ Parent-of-origin effects have also been observed on chromosomes 5p13 and 14q12 in relation to specific language impairment (SLI)^6^ and at the filaggrin gene (*FLG* [MIM: 135940]) on chromosome 1q21 in relation to childhood atopic dermatitis^7^ (although this *FLG* effect was interpreted as being most likely due to a direct effect of maternal genotype, rather than to differing effects of the alleles inherited from the different parents). Experimental studies investigating potential parent-of-origin effects detected at *COL2A1* (MIM: 120140) and *ABCA4* (MIM: 601691) in children with congenital toxoplaxmosis^8^ showed isoform-specific epigenetic modifications consistent with imprinting in both *COL2A1* and *ABCA4*.

A variety of statistical methods have been used for the detection and estimation of parent-of-origin effects in humans. We focus here on methods designed for binary (disease) traits, rather than on methods that have been developed for the analysis of quantitative traits.^9–12^ A review of the most popular currently used approaches is given by Connolly and Heron.^13^ One intuitive approach, available in the software package PLINK,^14^ is to use an adaptation of the transmission disequilibrium test (TDT),^15^ whereby transmissions and non-transmissions of an allele of interest to an affected offspring are stratified according to parental origin. However, such TDT-like approaches generally have the disadvantage of discarding observations in which both parents and offspring are heterozygous (given that parental origin cannot be assigned in this case), of erroneously assuming the transmissions from two heterozygous parents are independent (which is not true in the presence of child-genotype effects^16^), and of being sensitive to (i.e., invalid in the presence of) maternal-genotype effects. In an Icelandic study, Kong et al.^5^ used a case-control version of this TDT-like approach and overcame these limitations by performing long-range phasing of SNP data to infer haplotypes and comparing the resulting haplotypes with those present in the closest relatives on the paternal and maternal sides (considered as “surrogate parents”) in order to infer parent of origin; they also excluded the possibility of maternal-genotype effects by evaluating the effects of the non-transmitted maternal alleles.

Provided one is willing to assume an absence of maternal-genotype effects, valid parent-of-origin tests include the transmission asymmetry test (TAT),^17^ the (generally more powerful)^13^ parental asymmetry test (PAT),^16^ and the parent-of-origin-effects test statistic (POET).^18^ If one is not willing to make this somewhat restrictive assumption, likelihood ratio tests based on more complex log-linear,^17^ logistic,^16^ conditional logistic,^19^ or multinomial^20^ models are generally considered preferable.^13^ (Such approaches can also be implemented under the assumption of no maternal-genotype effects, to increase power, if desired.) A variety of software implementations for fitting these more complex models exists; Connolly and Heron^13^ recommend the use of our own software suite, PREMIM and EMIM,^20,21^ over other alternatives, on account of its ease of use for genome-wide data and generally high power for detection of parent-of-origin effects (even in the presence of other effects, such as those due to child or maternal genotype) while maintaining appropriate type I error rates. An additional attraction of PREMIM and EMIM is their ability to deal with data from either case-mother or case-father duos or case-parent trios, along with additional child and parent genotype data (such as that from individual case and control subjects or parents of case and control subjects) included when available. This allows the incorporation of families in which one (or more) individuals within a trio are missing, making maximum use of all available information.

Most existing methods for investigating parent-of-origin effects operate on a SNP-by-SNP basis, and each SNP is analyzed individually. When an ambiguous configuration with respect to parental origin is encountered at a SNP (such as observations in which both parents and an offspring are heterozygous), then either some sort of “averaging” over the possible parental transmissions is performed or else these ambiguous observations are discarded. In theory, greater information regarding parental origin could be obtained by considering several nearby SNPs simultaneously, as was done in the long-range phasing approach employed in the Icelandic study.^5^ Extensions to the PAT that take into account haplotypes of multiple tightly linked SNPs^22,23^ have demonstrated that increased power can indeed be obtained through this strategy, but these extensions suffer from the same problem as the original PAT of being sensitive to the presence of maternal-genotype effects. Gjessing and Lie^24^ present an extension to the log-linear modeling approach^17^ that models the effects of haplotypes defined by alleles at several nearby SNPs. Their extension is implemented in the software package HAPLIN. As pointed out by Shi et al.,^25^ estimation of risks for all haplotypes, as done by HAPLIN, becomes rapidly intractable with more than a few SNPs, on account of the fact that the number of possible haplotypes (and thus parameters to estimate) grows exponentially with the number of SNPs. Shi et al.^25^ propose their own haplotype-based extension to the log-linear modeling approach that uses the HAPLORE program^26^ to perform the initial haplotype estimation (phasing) step. Shi et al. achieve higher computational efficiency than Gjessing and Lie by focusing on candidate haplotypes that are nominated a priori based on prior knowledge. Although computationally convenient, this thus represents a somewhat restricted application. Modification of haplotype effects according to parental origin has also been incorporated in the UNPHASED software.^27^ However, UNPHASED, like HAPLIN, is limited (for computational reasons) to haplotypes comprised of no more than about five or six SNPs, meaning that a genome-wide analysis would need to be performed through repeated phasing across small sliding windows of haplotypes, a procedure that is both operationally and computationally inconvenient.

The issue of haplotype estimation (phasing) is by now quite well-studied in human genetics. Estimated haplotypes are routinely used for a variety of downstream analyses, including estimation of recombination rates,^28^ measurement of linkage disequilibrium,^29^ and genotype imputation.^30^ This desire to construct (potentially long-range) haplotypes, given unphased genotype data, has resulted in the development of a variety of software packages that can efficiently perform haplotype estimation on a chromosome-wide scale. Arguably one of the most competitive among these is the package SHAPEIT2,^31,32^ which has been found to outperform most other methods in terms of switch error rate and high computational efficiency.^32^ Although designed primarily for the analysis of unrelated individuals, SHAPEIT2 also has the advantage of being able to handle case-parent trios and duos. (These situations impose constraints on the configurations of possible haplotypes that are consistent within a duo or trio.) Given the availability of such a convenient software implementation for haploype phasing (in the form of SHAPEIT2), we sought to update our software suite, PREMIM and EMIM, to make use of haplotypes estimated with SHAPEIT2, in order to provide improved power for detection of parent-of-origin effects. Here, we present an overview and evaluation of our improved method, which has been incorporated into our freely available software package PREMIM and EMIM.^21^

## Material and Methods

For an overview of the methodology implemented in PREMIM and EMIM, see our previous work.^20,21^ Here, we shall describe only the essential components relevant to the current manuscript. EMIM uses genotype counts from pedigree data to estimate relative-risk parameters through the use of multinomial modeling. The accompanying program PREMIM pre-processes the pedigree data to supply EMIM with the required genotype count information. Parameters estimable by EMIM include child genotype effects *R*_1_ and *R*_2_ (the relative risks conferred by the presence of one or two copies of the risk allele in the child), maternal-genotype effects *S*_1_ and *S*_2_ (the relative risks conferred by the presence of one or two copies of the risk allele in the mother), and maternal and paternal parent-of-origin (or, imprinting) parameters *I*_*m*_ and *I*_*p*_, respectively, which correspond to the factor by which a child’s disease risk is multiplied if they inherit a risk allele from their mother or father. EMIM calculates a log likelihood at each SNP of interest, on the basis of the chosen parameters and assumptions, such as Hardy-Weinberg equilibrium or conditioning on parental genotypes.^21^

Here, we aim to increase the power to detect parent-of-origin effects by improving the information regarding the parental origin of a child’s alleles. First, we consider case-parent trios and then case-mother duos. By symmetry, all results for maternally inherited imprinting effects can be applied to paternally inherited imprinting effects, and, similarly, results for case-mother duos can be applied to case-father duos.

For case-parent trios, 15 possible genotype combinations (*g*_*m*_,*g*_*f*_,*g*_*c*_) can occur in a mother, father, and child at any given SNP (see Table 2 of Ainsworth et al.^20^). EMIM fits a multinomial model to the observed counts in these 15 categories. The only configuration in which it is not possible to determine the parental origin is when all three individuals are heterozygous. That is, if we denote the minor allele by “2” and the major allele by “1,” then if both parents and the child have genotype “1/2,” it is not known whether the “2” allele came from the father or the mother. (Throughout this paper we use “2” to denote the minor allele, which is also considered, for convenience, to be the risk allele, although in practice either allele can be modeled as the risk allele).

In previous versions of EMIM (≤ 2.07), the multinomial likelihood contribution from such a trio was

$$P\left(g_{m}=12,g_{f}=12,g_{c}=12\text{ or}\;\;21|\text{dis}=1\right)=\mu_{4}R_{1}S_{1}\left(I_{p}+I_{m}\right)\gamma_{11}$$

where dis = 1 denotes the event that the child is diseased, *g*_*c*_ denotes the ordered (maternal and paternal) alleles in the child, *μ*_4_ denotes a nuisance (mating-type stratification) parameter, and *γ*_11_ denotes an optional mother-child genotype-interaction parameter that can be estimated if desired. This likelihood contribution comes from combining into a single cell (cell 9) the contributions from cells 9a and 9b of Table 2 of Ainsworth et al.,^20^ in which cell 9a corresponds to the (unobservable) situation that the “2” allele in the child came from the father and 9b to the (unobservable) situation that the “2” allele in the child came from the mother.

In our updated version of EMIM, we use the software package SHAPEIT2^31,32^ to estimate haplotypes in the trio and then use this information to infer the parental origin of the “2” allele in the child. We thus consider cells 9a and 9b separately, resulting in a likelihood contribution of *μ*_4_*R*_1_*S*_1_*I*_*p*_*γ*_11_ if a trio is deemed to fall into category 9a or *μ*_4_*R*_1_*S*_1_*I*_*m*_*γ*_11_ if a trio is deemed to fall into category 9b. (Trios where there is still some ambiguity regarding parental origin could, in theory, contribute fractional counts to both cells; however, as noted later, we did not find any advantage in allowing for this as compared to just using the most likely parent-of-origin assignment).

Separation of cell 9 into two cells, 9a and 9b, leads to a situation in which the multinomial likelihood uses counts from 16 (rather than the originally considered 15) cells. However, we might want to analyze datasets in which only a proportion of the case-parent trios have been phased. This could occur, for example, if some of the case-parent trios have been genotyped only at a candidate SNP (so there are no surrounding SNPs to provide phase information), whereas other trios have been more densely genotyped. Thus, we actually need to consider the counts from 17 cells, with cells 9, 9a, and 9b all considered as separate categories. In Appendix A, we derive the multinomial likelihood that includes data for cells 9, 9a, and 9b in terms of the genotype relative-risk parameters of interest.

A similar approach can be used for case-mother or case-father duos. Table 3 of Ainsworth et al.^20^ shows the seven observable genotype combinations in case-mother duos (a similar table can be constructed for case-father duos). Here, cell 4 is the only configuration in which parental origin is not observed. Cell 4 can be divided into two cells: cell 4a, where the risk allele, “2,” is inherited from the father, and cell 4b, where it is inherited from the mother. In previous versions of EMIM (≤ 2.07), the multinomial likelihood contribution from cell 4 was *R*_1_*S*_1_*γ*_11_(*I*_*m*_(*μ*_4_ + *μ*_5_) + *I*_*p*_(*μ*_2_ + *μ*_4_)), whereas now this is separated out into two contributions, *R*_1_*S*_1_*γ*_11_(*I*_*p*_(*μ*_2_ + *μ*_4_)) for counts in cell 4a and *R*_1_*S*_1_*γ*_11_(*I*_*m*_(*μ*_4_ + *μ*_5_)) for counts in cell 4b. To allow for datasets in which only a proportion of the case-mother duos have been phased, we fit a likelihood to the counts from nine cells, with cells 4, 4a, and 4b all considered as separate categories (see Appendix A). A similar process can be carried out with the table for case-father duos. The overall likelihood of the data corresponds to the product of the likelihoods for the tables for different observed family units (including case-parent trios, case-mother duos, case-father duos, and various other case- and control-based tables, see Howey and Cordell^21^ for details).

### Workflow

The following steps are carried out when using PREMIM and EMIM in conjunction with SHAPEIT2 to analyze data from multiple nearby SNPs (including, but not limited to, genome-wide association study [GWAS] data). Note that steps 1–7 are carried out through a single command line call to PREMIM, which automatically invokes SHAPEIT2 as required.

1. PREMIM: Case-parent trios and duos are chosen from pedigrees. PREMIM processes the pedigree data and summarizes for each SNP the possible genotype combinations in case-parent trios and duos. The previous version of PREMIM processed pedigrees on a SNP-by-SNP basis so that the chosen case-parent trio (from a larger pedigree) for one SNP might be different from that for another SNP. For haplotype estimation, it is necessary that the same case-parent trio is chosen for every pedigree and SNP, so PREMIM chooses the case-parent trio with the least missing SNP data, but only if the amount of missing data is above a user-specified threshold (default, 50%). Following selection of case-parent trios, case-mother duos are next selected by PREMIM in the same manner except with the extra constraint that only pedigrees that have not had a case-parent trio selected are considered. The case-father duos are then selected with the constraint that only pedigrees that have not had a case-parent trio or case-mother duo selected are considered.
2. PREMIM: Binary pedigree files are created for case-parent trios and duos. The case-parent trios and duos selected for haplotype estimation are collected together into one PLINK^14^-format binary pedigree (.bed) file with associated family (.fam) and map (.bim) files.
3. SHAPEIT2: Haplotype graph is calculated. PREMIM invokes SHAPEIT2 to calculate the haplotype graph with data created in step 2. Several different options are available in SHAPEIT2 to try to improve the accuracy of the phasing at the cost of increasing the processing time. In our experience, we found that the default (slower) settings were best for duos, and thus these are used by default, but for trios these could be changed in order to speed up the processing time. It is also possible to use an external reference panel with SHAPEIT2, and to use a known recombination map, which might be beneficial for some datasets. However, in our experience, case-parent trios and duos generally provide sufficient information for excellent resolution of parental origin even without making use of a reference panel or a known recombination map. SHAPEIT2 also imputes missing data and handles Mendelian errors by setting genotypes to missing.
4. SHAPEIT2: Haplotypes are estimated. PREMIM then evokes SHAPEIT2 to return the most probable haplotype estimates from the haplotype graph calculated in step 3. It was found (data not shown) that allowing for phase uncertainty through sampling possible haplotypes from the haplotype graph did not improve performance in terms of power or type I error, although in theory this could be done (with the results averaged to generate non-integer cell counts for cells 9a and 9b or for cells 4a and 4b) if desired.
5. PREMIM: Phased case-parent trio and duo data processed. PREMIM estimates the parent of origin of alleles for ambiguous scenarios by using the phased haplotypes from SHAPEIT2. The total counts for trios and duos that are phased and not phased are also recorded for each SNP and are used to calculate the likelihood. The resolution of ambiguous trios and duos is recorded as cell counts 9a and 9b for case-parent trios and cell counts 4a and 4b for duos.
6. PREMIM: Phased duo data are adjusted. The estimated counts in cell 4a and 4b for duos have been found to sometimes lead to an inflated test statistic in EMIM. Therefore, these counts are adjusted to reduce the inflation to an acceptable level, see Appendix B for details.
7. PREMIM: Remaining pedigrees are processed. Any pedigrees without a case-parent trio or duo selected for phasing are processed by PREMIM in the usual manner on a SNP-by-SNP basis, possibly creating other pedigree subunits, such as parents of a case subject, lone case subjects, and control subjects. Each pedigree subunit has a file created with the genotype counts for each SNP. Any case-parent trio or duo data processed without phasing is combined with the phased data to create EMIM input files with counts in all three relevant categories (9, 9a, and 9b for trios or 4, 4a, and 4b for duos).
8. EMIM: Case-parent trio and duo data are analyzed together with other pedigree data. The genotype count files created by PREMIM are analyzed by EMIM (with a slightly updated-format parameter file that specifies the parameters to estimate and the model assumptions).

### Adjustment of Genotype Counts for Duos

Initial investigations indicated that for ambiguous duos (in which the parent and child are both heterozygous), when the number of minor alleles inherited from the father and mother were estimated with SHAPEIT2, the estimates could be biased, depending on the minor allele frequency and which parent (mother or father) was genotyped, leading to inflated test statistics in EMIM. To correct this bias, we devised an adjustment procedure that relies on the fact that we will have tested many SNPs, most of which will not display parent-of-origin effects. (Our adjustment is thus suitable for GWAS data or data from a set of SNPs that are not expected to show parent-of-origin effects; it would not be suitable for analyzing a small number of candidate SNPs.) See Appendix B and Figure S1 for details and an example of the proposed adjustment procedure. Our adjustment procedure involves fitting curves to the estimated counts that correspond to adjusted versions of the curves expected under the null hypothesis. The fitted curves include an adjustment function, *f*(*p*), where *p* is the minor allele frequency. The cell counts for minor alleles inherited from the father (cell 4a) and mother (cell 4b) are then adjusted by respectively subtracting and adding *f*(*p*). (This can result in non-integer values for the adjusted counts, which is not a problem given that the multinomial likelihood maximized by EMIM does not specifically require the counts to be integers, see Appendix A.) This procedure ensures that, for the adjusted counts, there should, on average, be far less bias toward transmissions being estimated as coming from one particular parent. A particular SNP that displays clear evidence of transmission from one parent rather than from another, as expected if genuine parent-of-origin effects exist, will, however, be only marginally affected by this adjustment, given that the vast majority of SNPs are assumed to be non-causal. It will be shown later (see Results) that this reduces inflation of the test statistic and slightly increases the power.

### Simulations for Investigating Power and Type I Error

We carried out a simulation study to investigate the performance of our proposed new approach. SimPed^33^ was used to simulate 1,000 (for investigation of power) or 5,000 (for investigation of type I error) replicates of datasets, each with 1,500 family units (case-parent trios, case-mother duos, or case-father duos) typed at 200 SNPs across a “chromosome.” Haplotype blocks of eight SNPs in length were simulated; this was repeated 25 times to give the total of 200 SNPs. If a causal SNP was required (as when estimating power), then the 100^th^ SNP was used. The power or type I error of PREMIM and EMIM under various models was then calculated; detection at SNP numbers 97 to 104 was used as evidence of a true or false finding. Several different PREMIM and EMIM tests were considered: (1) using the parent of origin of alleles as estimated from SHAPEIT2, with and without genotype-count adjustment, (2) using the previous version of PREMIM and EMIM, which categorizes ambiguous trios or duos into a single cell (cell 9 for trios or cell 4 for duos) without estimating parent of origin, and (3) using the known (simulated) parent of origin of alleles. The p value thresholds used to examine power were 10^−12^, 10^−10^, and 10^−6^. For type I error, the p value thresholds used were 6.25 × 10^−3^, 1.25 × 10^−3^, and 1.25 × 10^−4^, which correspond to family-wise error rates (FWERs) of 0.05, 0.01, and 0.001, under the assumption that the eight SNPs tested are independent. Unless otherwise stated, the default options in SHAPEIT2 were used (“−burn 7 −prune 8 −main 20”). For faster analysis (used in the simulation study for case-parent trios), SHAPEIT2 with fast MCMC options (“−burn 1 −prune 1 −main 1”) was used. Tests were performed with PREMIM and EMIM to detect (1) maternally inherited imprinting effects, (2) maternally inherited imprinting effects while allowing for child effects, and, for case-parent trios and type I errors only, (3) maternally inherited imprinting effects while allowing for maternal effects and (4) maternally inherited imprinting effects while allowing for maternal and child effects.

### Application to SLI Data

SLI is a neurodevelopmental disorder that affects linguistic abilities when development is otherwise normal. In a recent GWAS of 297 affected children in 278 pedigrees, Nudel et al.^6^ found two chromosomal regions of interest: chromosome 14, with a paternally inherited parent-of-origin effect (*I*_*p*_, p value = 3.74 × 10^−8^) and chromosome 5, with a maternally inherited parent-of-origin effect (*I*_*m*_, p value = 1.16 × 10^−7^). We applied the latest versions of PREMIM and EMIM (using SHAPEIT2 to estimate the parent-of-origin of alleles) to a slightly updated version of this SLI dataset, testing for paternally inherited parent-of-origin effects on chromosome 14 and maternally inherited parent-of-origin effects on chromosome 5. The pedigrees were subjected to quality control measures as described in Anderson et al.^34^ and Nudel et al,^6^ but note that the threshold used for exclusion on the basis of heterozygosity rates was ± 3 SD from the mean, and the Hardy-Weinberg equilibrium p value exclusion threshold used in PLINK was 10^−6^ (and not ± 2 SD and 0.001, respectively, as previously incorrectly specified in Nudel et al.^6^).

### Application to Tetralogy of Fallot Data

Tetralogy of Fallot (TOF) is the most common form of congenital heart disease, a major source of morbidity and mortality in childhood. In a recent GWAS using a European discovery set of 835 case subjects, 717 additional family members (including both parents for 293 of the case subjects), and 5,159 control subjects, Cordell et al.^35^ found regions on chromosomes 12 and 13 to be significantly and replicably associated with TOF. Although not reported by Cordell et al.,^35^ further modeling of the replicating regions via EMIM indicated that the top result on chromosome 12 (at rs11065987) could be equally well modeled by a paternally inherited imprinting effect (*I*_*p*_, p value = 2.10 × 10^−8^) as by an allelic effect of a child’s own genotype (p value = 4.06 × 10^−8^). Also, the top result on chromosome 13 (at rs7982677) could potentially be equally well modeled by a maternally inherited imprinting effect (*I*_*m*_, p value = 9.54 × 10^−7^) as by an allelic effect of a child’s own genotype (p value 6.41 × 10^−7^), although there wasn’t sufficient power in either case to distinguish between imprinting and child-genotype effects. Evidence for a maternally inherited imprinting effect on chromosome 12 or a paternally inherited imprinting effect on chromosome 13 was less well supported (*I*_*m*_, p value = 9.04 × 10^−5^ at rs11065987; *I*_*p*_, p value 0.00022 at rs7982677). Here, we investigate these findings further by using our updated version of PREMIM and EMIM, which uses SHAPEIT2 to estimate the parent-of-origin of alleles, to test for paternally inherited imprinting effects on chromosome 12 and maternally inherited imprinting effects on chromosome 13.

## Results

Here, we present results using the latest versions of our programs PREMIM and EMIM,^21^ which have been updated to incorporate haplotype estimation when modeling parent-of-origin effects. PREMIM calls (if requested) the software package SHAPEIT2,^31,32^ and EMIM then incorporates the estimates of parent of origin of alleles provided by SHAPEIT2 into its own multinomial modeling procedure to increase power.

### Power for Case-Parent Trios

**Figure 1:**
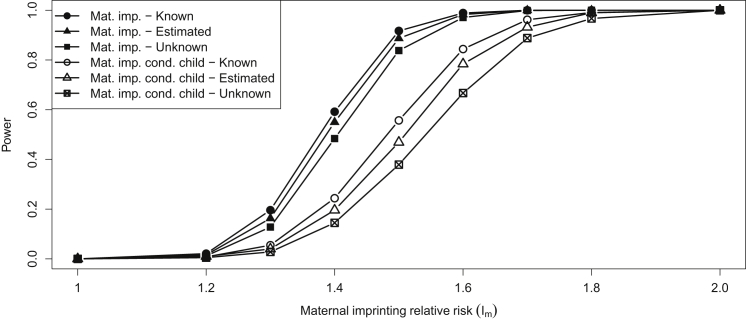
Power of EMIM when Using Simulated Case-Parent Trio Data Powers of EMIM (for p value threshold 10^−6^) when using simulated case-parent trio data and assuming known parent of origin, parent of origin estimated with haplotype estimates from SHAPEIT2, or unknown (so, averaged over) parent of origin for any ambiguous trios. Results are shown for tests of both maternally inherited imprinting effects and maternally inherited imprinting effects conditional on child-genotype effects.

The power to detect a maternally inherited imprinting effect (i.e., in which the allele inherited from the mother increases disease risk) with 1,500 simulated case-parent trios is shown in Figure 1. Powers are presented for varying values of the imprinting parameter *I*_*m*_ (representing the risk factor conferred by the maternal risk allele) with or without additionally conditioning on (i.e., allowing for) child-genotype effects. In each case, the top line shows the power of EMIM when using known parent of origin (calculable here because this is simulated data), the middle line shows the power of EMIM when using haplotype estimation in SHAPEIT2, and the lower line shows the power when using the previous version of EMIM. It can be seen that haplotype estimation increases the power and provides a level of power that is not too far from the maximum power achievable when using the known parent of origin of alleles.

**Figure 2:**
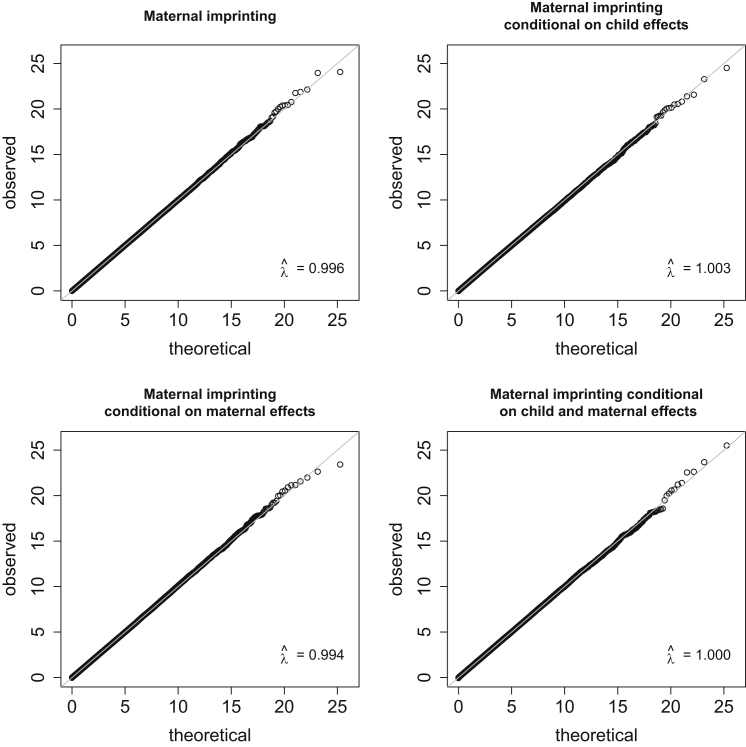
Q-Q Plots for Simulated Case-Parent Trio Data Q-Q plots showing observed test statistics against their theoretical values for simulated case-parent trio data when using EMIM with SHAPEIT2 to test (1) maternally inherited imprinting effects, (2) maternally inherited imprinting effects conditional on child-genotype effects, (3) maternally inherited imprinting effects conditional on maternal-genotype effects, and (4) maternally inherited imprinting effects conditional on child and maternal effects. $\hat{\lambda }$ indicates the genomic control inflation factor.

We investigated the use of different options within SHAPEIT2 to try to improve the accuracy of estimation of haplotypes and thus of parent of origin. For trios, it was found that no options provided higher power than using the most basic Markov chain Monte Carlo (MCMC) options in SHAPEIT2 (“−burn 1 −prune 1 −main 1”) and the default options for the size of the SNP window (2Mb) and the states (“−window 2 −states 100”) (results not shown), and these options provided adequate control of type I error (Figure 2, Figure S2). We also investigated sampling from the posterior distribution of haplotypes within SHAPEIT2 in order to allow for haplotype uncertainty, but this did not improve either power or type I error in comparison to just using the most likely haplotype configuration (results not shown).

The type I error obtained with the new versions of PREMIM and EMIM was compared with that obtained with previous versions of PREMIM/EMIM and with that obtained when parent of origin is known. All three approaches gave the same levels of type I error and no signs of inflation (Figure S2). Quantile-quantile (Q-Q) plots of the test statistics from the new versions of PREMIM and EMIM (derived from 5,000 simulation replicates under the null hypothesis, each consisting of 1,500 case-parent trios and 200 SNPs) are shown in Figure 2. The results shown are for tests of maternally inherited imprinting effects, maternally inherited imprinting effects conditional on child-genotype effects, maternally inherited imprinting effects conditional on maternal-genotype effects, and maternally inherited imprinting effects conditional on both child and maternal effects. The observed genomic control^36^ inflation factors are all approximately 1.0, as expected for a well-calibrated test.

### Power for Case-Mother Duos

**Figure 3:**
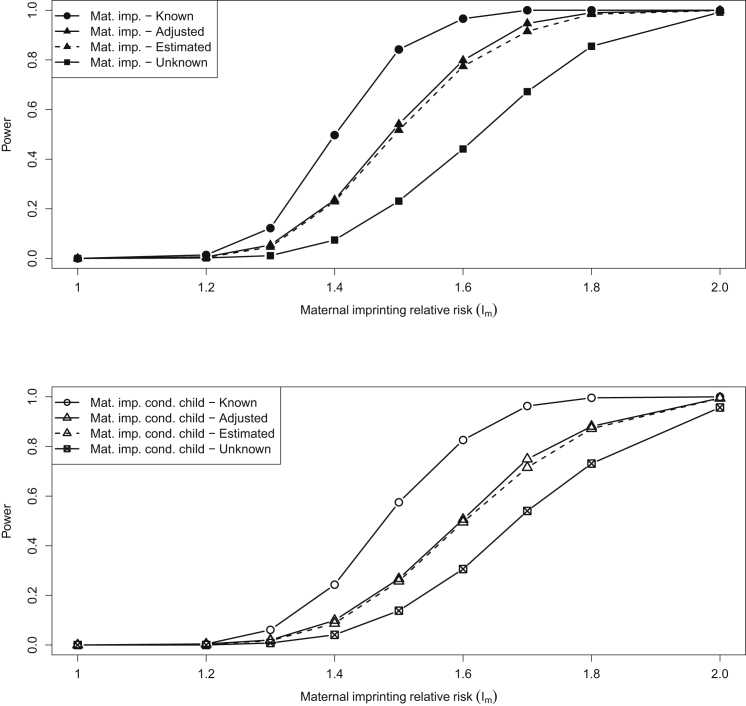
Power of EMIM when Using Simulated Case-Mother Duo Data Powers of EMIM (for p value threshold 10^−6^) when using simulated case-mother duo data and assuming known parent of origin, parent of origin estimated with haplotype estimates from SHAPEIT2, or unknown (so, averaged over) parent of origin for any ambiguous duos. Tests of maternally inherited imprinting effects are shown on the top plot and maternally inherited imprinting effects conditional on child effects are shown on the lower plot.

In many studies, genotype data for case subjects and their mothers are available, but no genotype information is available for the father. We used the same approach as used above for case-parent trios to investigate the powers to detect maternally inherited imprinting effects and maternally inherited imprinting effects conditional on child effects, given genotype data for 1,500 case-mother duos. Results are shown in Figure 3. In general, the powers are seen to be lower than when using case-parent trios because there is less information available to determine the parent of origin of the child’s alleles.

The top lines of both plots in Figure 3 show the power of EMIM when using known parent of origin (calculable here because this is simulated data). It is interesting to note that the powers are slightly less than those seen with case-parent trios, even though in both scenarios we have the same number of cases and perfect information on the parent of origin of the alleles of interest. This loss of power is most likely due to there being less information available to estimate the nuisance parameters (the minor allele frequency and the parental mating parameters *μ*_1_,…,*μ*_6_, see Ainsworth et al.^20^).

The middle lines of both plots in Figure 3 show the power of EMIM when incorporating haplotype estimation, with the cell counts either unadjusted or adjusted by PREMIM (see Material and Methods for description of adjustment procedure). It can be seen that the use of estimated parent of origin through haplotype estimation provides a substantial increase in power in comparison to that of the previous version of EMIM (the bottom line of both plots), which effectively “averages” over parent of origin. The greater improvement in power from haplotype estimation seen for case-mother duos in comparison to that for case-parent trios is due to the larger proportion of families in which the parent of origin of alleles is ambiguous (for case-mother duos, only the mother and child must be heterozygous for parent of origin to be unobserved, whereas for case-parent trios, all three individuals must be heterozygous). The power when using adjusted estimates (see Material and Methods) shows a slight improvement over the power when using unadjusted estimates, owing to the fact that the estimation of counts of duos falling into the different parent-of-origin categories is improved through the adjustment procedure.

**Figure 4:**
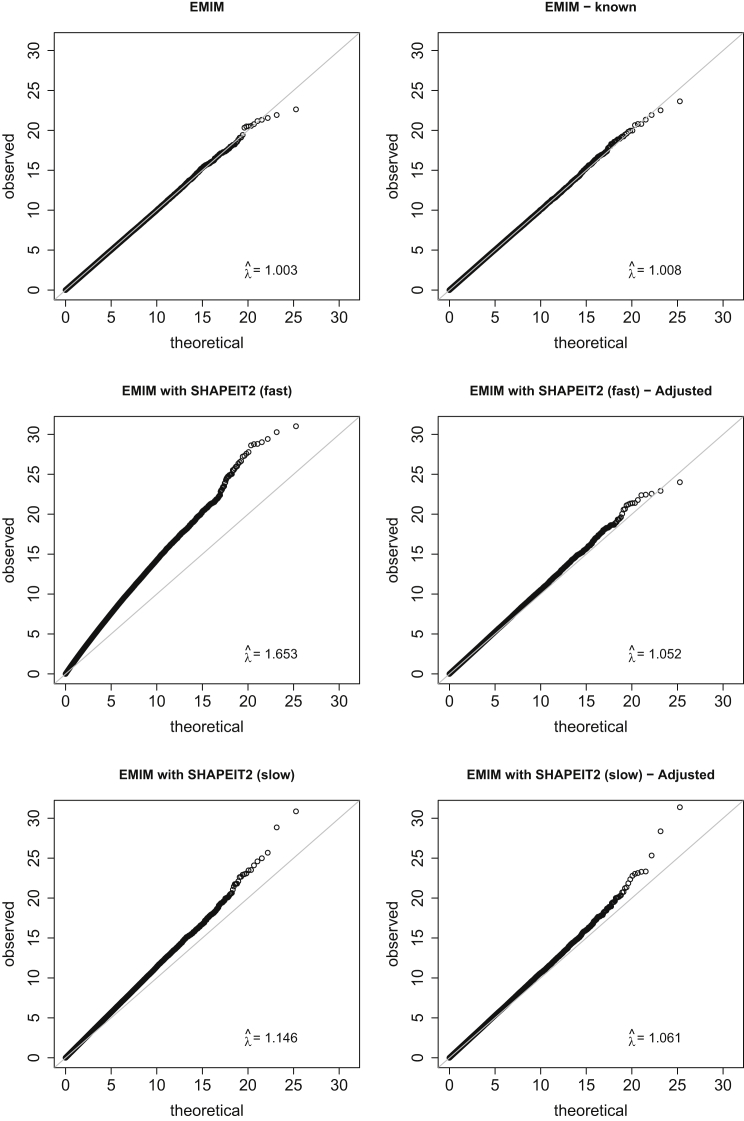
Q-Q Plots for Simulated Case-Mother Duo Data Q-Q plots showing observed test statistics against their theoretical values for simulated case-mother duo data when using EMIM to detect maternally inherited imprinting effects in 1,500 case-mother duos with (1) EMIM alone, (2) EMIM with known parent-of-origin alleles, (3) EMIM using SHAPEIT2 with fast MCMC parameters, (4) EMIM using SHAPEIT2 with fast MCMC parameters and adjusted counts, (5) EMIM using SHAPEIT2 with slow MCMC parameters, and (6) EMIM using SHAPEIT2 with slow MCMC parameters and adjusted counts. $\hat{\lambda }$ indicates the genomic control inflation factor.

Figure S3 shows the type I error of the different EMIM tests, and dashed lines show the expected FWERs if the eight SNPs in the simulated haplotype are (conservatively) considered to be independent. The type I error of the previous version of EMIM (labeled “unknown”), which has been extensively evaluated by Ainsworth et al.,^20^ acts as a guide to the “correct” type I error rate, allowing for dependency between the SNPs. The type I error when using SHAPEIT2 with fast parameter options is very inflated. This is illustrated further in the Q-Q plots (Figure 4) in which the genomic control inflation factor *λ* = 1.653. However, the inflation factor can be reduced to 1.052 when using PREMIM’s adjustment procedure. The improvement obtained from adjustment when using the default (slower) parameter options in SHAPEIT2 is not as pronounced: the inflation factor is 1.146 for the unadjusted analysis and reduces to 1.061 when the counts are adjusted (see Figure 4).

### Utility of Paternal Data for Detection of Maternally Inherited Imprinting Effects

Although case-mother duos are a more commonly used unit than case-father duos, in real studies, both types of duos might be collected. In Figure S4, it can be seen that, somewhat counter-intuitively, when performing a test of the parameter *I*_*m*_ in the presence of genuine maternally inherited imprinting effects, more power is gained from a sample of case-father duos than from a sample of case-mother duos. (Both types of duos provide less power than case-parent trios.) This is true whether or not haplotype estimation with SHAPEIT2 is performed. This observation can be explained by the observation that, to detect maternally inherited imprinting effects, we are only interested in determining whether or not the child inherited a risk allele from the mother. The expected proportion of case-mother duos in which the parent of origin can be determined unambiguously can be calculated from column five of Table 3 of Ainsworth et al.;^20^ a similar calculation can be performed for case-father duos. Performing this calculation, we find that, provided the allele frequency of the maternally transmitted allele that increases disease risk is < 0.5 (i.e., the “risk” allele is the minor allele), a higher proportion of case-father duos than case-mother duos allows unambiguous determination of the parent of origin (data not shown). If the major allele is the risk allele, this is reversed, and it is the case-mother duos that provide the greater power. Similarly, for a paternally inherited imprinting effect, provided the risk allele is the minor allele, it is the case-mother duos that provide the higher power; if instead the risk allele is the major allele, case-father duos provide higher power.

### SLI Data

**Figure 5:**
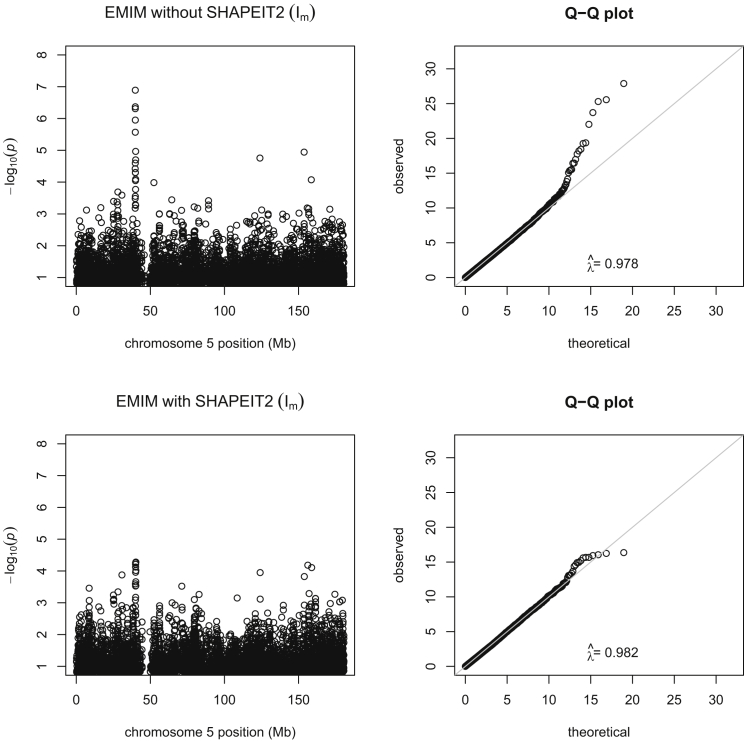
Manhattan and Q-Q Plots for Specific Language Impairment Data, Chromosome 5 Manhattan plots of the −log_10_ p values and Q-Q plots of the test statistics on chromosome 5 for maternally inherited imprinting effect analysis of specific language impairment data when using EMIM alone (top plots) and EMIM with SHAPEIT2 (bottom plots). $\hat{\lambda }$ indicates the genomic control inflation factor.

We re-analyzed an updated version of the dataset of Nudel et al. (2014),^6^ who had presented evidence for a maternally inherited parent-of-origin effect on chromosome 5 and a paternally inherited parent-of-origin effect on chromosome 14. Re-analysis of the chromosome 5 data in PREMIM and EMIM without using haplotype estimation in SHAPEIT2 gave a minimum p value of 1.29 × 10^−7^ at rs10447141, very similar to that seen in the original analysis by Nudel et al. (p value = 1.16 × 10^−7^). Using PREMIM and EMIM with haplotype estimation in SHAPEIT2 gave a less significant p value of 6.18 × 10^−5^ at the same SNP. Plots of the two analyses, with and without use of SHAPEIT2 to estimate the parent of origin of alleles, are shown in Figure 5 and show a considerable decrease in significance of the most significant p values. The p values in the implicated region when using estimated parent of origin now provide only weak evidence of association as a result of a maternally inherited imprinting effect.

**Figure 6:**
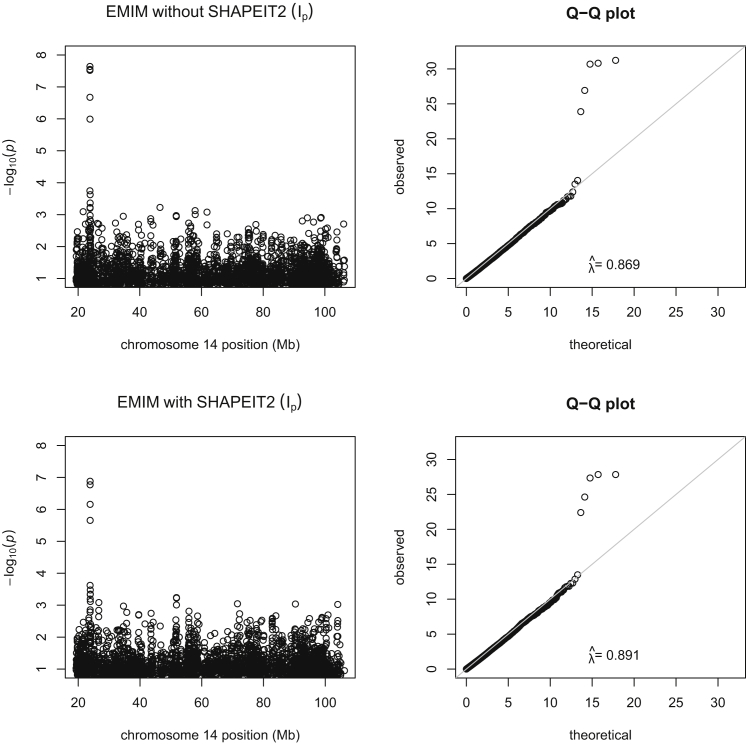
Manhattan and Q-Q Plots for Specific Language Impairment Data, Chromosome 14 Manhattan plots of the −log_10_ p values and Q-Q plots of the test statistics on chromosome 14 for paternally inherited imprinting effects analysis of specific language impairment data when using EMIM alone (top plots) and EMIM with SHAPEIT2 (bottom plots). $\hat{\lambda }$ indicates the genomic control inflation factor.

Re-analysis of the SLI data on chromosome 14 without use of haplotype estimation in SHAPEIT2 gave a minimum p value of 2.29 × 10^−8^ at rs4280164, very similar to that seen in the original analysis by Nudel et al. (p value = 3.74 × 10^−8^). Using PREMIM/EMIM with haplotype estimation in SHAPEIT2 gave a less significant p value of 1.32 × 10^−7^ at the same SNP. Plots of the two analyses, with and without estimated parent of origin, are shown in Figure 6. These plots show a general decrease in significance of the most significant p values when using haplotype estimation in SHAPEIT2, although the results (see bottom plots of Figure 6) do still provide positive evidence of association due to a paternally inherited imprinting effect.

To investigate the cause of the decreases in significance seen in the SLI study when incorporating haplotype estimation, we examined the counts of trios and duos falling into the various categories used in EMIM’s multinomial modeling procedure (see Table 1). Cells 9 (for trios) or 4 (for duos) correspond to the ambiguous categories in which all individuals are heterozygous; when using haplotype estimation in SHAPEIT2, these cells are decomposed into cells 9a and 9b, or 4a and 4b, respectively, in which parent of origin has been (probabilistically) determined. For SNP rs10447141 on chromosome 5, there are 16 ambiguous case-parent trios, which result (when using haplotype estimation) in estimates of six case subjects receiving the risk allele from the father and ten from the mother. There were also ten ambiguous case-mother duos, resulting in estimates of 1.99 case subjects receiving the risk allele from the father and 8.01 from the mother, and four ambiguous case-father duos, resulting in estimates of 3.02 cases receiving the risk allele from the father and 0.98 from the mother. Overall, this resolves the parent of origin in an additional 30 families in comparison to the original analysis of Nudel et al.,^6^ giving 11.01 new receipts from the father and 18.99 from the mother. This increased number of receipts from the mother might be expected to result in a stronger maternally inherited imprinting effect than seen originally; however, the “risk” allele in this case actually decreases risk, giving an odds ratio of *I*_*m*_ = 0.326 in the original analysis and *I*_*m*_ = 0.494 when using estimated parent of origin. Therefore, the overall effect is now weakened by having more new receipts from mothers than from fathers. Similar observations can be made for the other SNPs in this region. It therefore seems probable that the original result could represent a statistical false positive due to stochastic sampling variation, which has been better resolved with the addition of 30 new observations. So, although the overall decrease in significance might seem disappointing, it corresponds to the use of more information (an additional 30 families contributing to the analysis) and thus should be considered a more reliable result.

The reduction in significance for the paternally inherited imprinting effect on chromosome 14 can also be explained by examining the parent-of-origin resolved cell counts (7.01 new receipts from the father and 15.99 from the mother, see Table 1). The fact that an additional 23 families are contributing to the analysis means that this should again be considered a more reliable result in comparison to the original analysis. However, in this case, the evidence for the presence of a paternally inherited imprinting effect remains relatively strong (p value = 1.32 × 10^−7^).

### TOF Data

We also re-analyzed the data of Cordell et al. (2013),^35^ testing for a paternally inherited imprinting effect on chromosome 12 and a maternally inherited imprinting effect on chromosome 13. Without using estimated parent of origin, the analysis on chromosome 12 gave a minimum p value of 2.10 × 10^−8^ at rs11065987; using estimated parent of origin gave a p value of 4.16 × 10^−7^ at the same SNP. On chromosome 13, the most significant SNP, rs7982677, gave p values of 9.54 × 10^−7^ and 6.97 × 10^−6^ when not estimating and estimating parent of origin, respectively. Plots of these analyses, with and without using estimated parent of origin, are shown in Figures S5 and S6. Again we see a general decrease in the significance of the most significant results when using haplotype estimation, weakening the evidence that these genomic regions genuinely harbor imprinting effects.

Table 2 shows counts of the number of trios or duos falling into the ambiguous categories and their resolution when using haplotype estimation with SHAPEIT2 for the TOF data. For SNP rs11065987 on chromosome 12, it can be seen that, for case-parent trios and case-father duos, the number of risk alleles inherited from the father and mother are approximately equal; however, for case-mother duos, many more risk alleles are estimated to be inherited from the mother: 10.007, as compared to 1.993 from the father. This results in the initial estimated paternally inherited imprinting effects odds ratio *I*_*p*_ = 1.662 reducing to 1.555, thus decreasing the significance (and increasing the p value). Similarly, for SNP rs7982677 on chromosome 13, it can be seen that more alleles are inherited from the father than from the mother for all trios and duos, resulting in an initial estimated maternally inherited imprinting effect odds ratio *I*_*m*_ = 1.577 reducing to 1.504. Again, although the significance is decreased when using haplotype estimation, it corresponds to the use of more information (an additional 43 families on chromosome 12 and an additional 29 families on chromosome 14) and thus should be considered the more reliable result. We note that the decrease of significance seen in this dataset with respect to testing imprinting effects (*I*_*p*_ or *I*_*m*_) has no impact on the significance of associations due to the case subject’s own genotype, findings which have in any case already been replicated in independent cohorts.^35^

## Discussion

Here, we have demonstrated the improved functionality implemented in the latest version of our software package, PREMIM and EMIM, which has been updated to incorporate haplotype estimation in SHAPEIT2 when modeling parent-of-origin effects. Naturally, computation time is increased as a result of using SHAPEIT2, but the analysis still remains quite feasible for any realistically sized study. Although the focus of this current investigation is on detection of parent-of-origin effects, we note that PREMIM and EMIM offer wider functionality, such as the ability to test for maternal-genotype effects or mother-child genotype interactions, with or without the inclusion of parent-of-origin effects. The software can also be used to increase the power when analyzing an unrelated case-control dataset by incorporating case or control parental data when available.

The ability to detect imprinting effects is dependent on determining the parent of origin of alleles in a set of case subjects, which requires parental data as well as data from the case subjects themselves. The most obvious data to gather is a case-parent trio so that the parent of origin of alleles can be determined, except in the one scenario where all individuals are heterozygous. With the use of SHAPEIT2, the parent of origin of alleles can be estimated by using the surrounding SNP information to estimate haplotypes and thus parent of origin. By using these estimates in our multinomial modeling procedure, we found that the power could be increased over the previous versions of PREMIM and EMIM, while still retaining acceptable type I error rates.

When data are restricted to case-mother or case-father duos, the proportion of genotype data with ambiguous parent of origin is greater than that for case-parent trios, thus greatly increasing the benefit of using SHAPEIT2 to estimate the parent of origin of alleles. However, use of case-mother or case-father duos with estimated haplotypes was found to lead to a potential bias in the parent-of-origin assignment (the bias was dependent on which parent was genotyped and the minor allele frequency of the SNP being tested), resulting in an increase in type I error. Fortunately, this could be corrected by adjusting the estimated parent-of-origin cell counts to more closely match the expected distributions under the null hypothesis. This adjustment was performed by fitting an adjustment curve via maximum likelihood methods, which successfully reduced the type I errors to acceptable levels as well as increased the power.

Parent-of-origin effects, particularly if mediated through imprinting, represent a more complex, potentially functionally relevant mechanism than the genetic effects that are typically identified through large-scale case-control GWASs. The requirement for parental data necessarily limits the power of studies designed to detect such effects (except, perhaps, in special populations such as the Icelandic population), owing to the decreased sample sizes that are likely to be available; however, suitable cohorts (particularly of mother-child duos) are often collected, for example, when investigating traits related to pregnancy complications. An attraction of focusing on the detection of parent-of-origin effects (rather than on effects mediated primarily by the case subject’s own genotype) is the greater potential for immediate functional investigation and experimental validation. Our application of the new versions of PREMIM and EMIM to four genomic regions that have been postulated as harboring parent-of-origin effects (in cohorts with two separate disorders) found slightly decreased evidence in three of the four regions and considerably decreased evidence in one region, as a result of a larger number of parent-of-origin resolved transmissions than had previously been available. None of the SNPs investigated lie in regions already known to contain imprinted genes,^37,38^ so further investigation will be required to determine the underlying cause of the parent-of-origin effects observed. The parent-of-origin effect seen on 14q for SLI does, however, overlap with a region that has previously shown a hint toward genomic imprinting.^39^ A comparison of individuals with a paternal deletion of 14q11–13 and individuals with maternal uniparental disomy of that region showed that the two groups had some overlapping phenotypes, suggesting that the paternal allele is normally expressed.^40^ Together, these studies suggest that the region may be maternally imprinted, and thus paternal parent-of-origin effects may be operating there.

In conclusion, we recommend that investigators interested in analyzing GWAS data to search for maternally or paternally inherited imprinting effects should use the updated approach implemented in our software package PREMIM and EMIM, which currently provides, to the best of our knowledge, the most convenient and powerful analysis tool for addressing this question.

## Figures and Tables

**Table 1:** Cell Counts for the Specific Language Impairment Data

**Chromosome**	**SNP**	**Base-Pair Position**	**Cell Counts in Case-Parent Trios**			**Cell Counts in Case-Mother Duos**			**Cell Counts in Case-Father Duos**			**EMIM Odds Ratio with Parent of Origin**		**EMIM p Value with Parent of Origin**	
**9**	**9a**	**9b**	**4**	**4a**	**4b**	**4**	**4a**	**4b**	**Unknown**	**Estimated**	**Unknown**	**Estimated**
5	rs10447141	39852924	16	6	10	10	1.99	8.01	4	3.02	0.98	0.326	0.494	1.29 × 10^−7^	6.18 × 10^−5^
5	rs980306	39852592	14	6	8	9	1.99	7.01	4	3.02	0.98	0.334	0.487	4.28 × 10^−7^	7.53 × 10^−5^
5	rs17194068	39857074	14	6	8	9	1.99	7.01	4	3.02	0.98	0.336	0.487	4.91 × 10^−7^	7.53 × 10^−5^
5	rs6895329	39861497	15	6	9	11	2.99	8.01	4	3.02	0.98	0.343	0.497	1.12 × 10^−6^	1.10 × 10^−4^
5	rs1994882	39841921	7	7	0	13	1.99	11.01	2	1.02	0.98	2.388	2.013	2.70 × 10^−6^	5.57 × 10^−5^
14	rs4280164	23841124	10	5	5	12	1.01	10.99	1	1.00	0.00	0.254	0.317	2.29 × 10^−8^	1.32 × 10^−7^
14	rs11158632	23839502	10	5	5	12	1.01	10.99	1	1.00	0.00	0.256	0.320	2.83 × 10^−8^	1.70 × 10^−7^
14	rs2144494	23843226	10	5	5	12	1.01	10.99	1	1.00	0.00	0.256	0.317	3.03 × 10^−8^	1.31 × 10^−7^
14	rs2281472	23845685	12	6	6	10	1.02	8.98	2	2.00	0.00	0.299	0.364	2.12 × 10^−7^	6.96 × 10^−7^
14	rs3181384	23856815	11	5	6	11	1.02	9.98	1	1.00	0.00	0.326	0.376	1.02 × 10^−6^	2.21 × 10^−6^

Shown are the results at the five SNPs on chromosomes 5 and 14 with the lowest p values, when using EMIM with and without haplotype estimation. Cell counts are shown for the ambiguous scenarios in which all individuals are heterozygous: cell 9 for case-parent trios and cell 4 for case-mother duos and case-father duos. The estimated number of trios and duos in which the risk allele is inherited from the father is given as cells 9a and 4a, respectively, and the estimated number in which the risk allele is inherited from the mother is given as cells 9b and 4b, respectively. Cell counts estimated from duos need not be integers as they incorporate the adjustment described in Appendix B. The odds ratios and p values given by EMIM with and without haplotype estimation are shown. Chromosomes 5 and 14 were tested for maternally and paternally inherited imprinting effects, respectively.

**Table 2:** Cell Counts for the Tetralogy of Fallot Data

**Chromosome**	**SNP**	**Base-Pair Position**	**Cell Counts in Case-Parent Trios**			**Cell Counts in Case-Mother Duos**			**Cell Counts in Case-Father Duos**			**EMIM Odds Ratio with Parent of Origin**		**EMIM p Value with Parent of Origin**	
**9**	**9a**	**9b**	**4**	**4a**	**4b**	**4**	**4a**	**4b**	**Unknown**	**Estimated**	**Unknown**	**Estimated**
12	rs11065987	110556807	28	13	15	12	1.993	10.007	3	1.976	1.024	1.662	1.555	2.10 × 10^−8^	4.16 × 10^−7^
12	rs11066188	111095097	30	13	17	13	2.996	10.004	4	2.976	1.024	1.591	1.489	3.38 × 10^−7^	5.02 × 10^−6^
12	rs3184504	110368991	30	14	16	11	9.942	1.058	3	0.987	2.013	0.622	0.662	3.43 × 10^−7^	3.35 × 10^−6^
12	rs17696736	110971201	29	13	16	12	1.987	10.013	4	2.977	1.023	1.582	1.481	4.63 × 10^−7^	6.84 × 10^−6^
12	rs653178	110492139	30	14	16	11	9.943	1.057	3	0.987	2.013	0.627	0.666	4.82 × 10^−7^	4.50 × 10^−6^
13	rs7982677	91786324	19	10	9	6	3.016	2.984	4	4.000	0.000	1.577	1.504	9.54 × 10^−7^	6.97 × 10^−6^
13	rs7318834	108710500	11	5	6	6	1.006	4.994	1	0.987	0.013	1.667	1.640	5.87 × 10^−6^	8.40 × 10^−6^
13	rs4771856	91792510	18	9	9	6	3.016	2.984	3	2.998	0.002	1.519	1.461	1.12 × 10^−5^	4.79 × 10^−5^
13	rs7994141	47672251	42	24	18	4	2.008	1.992	4	2.007	1.993	0.656	0.668	1.41 × 10^−5^	1.13 × 10^−5^
13	rs7995410	47651319	42	24	18	4	2.008	1.992	4	2.007	1.993	0.656	0.668	1.43 × 10^−5^	1.14 × 10^−5^

Shown are the results at the five SNPs on chromosomes 12 and 13 with the lowest p values, when using EMIM with and without haplotype estimation. Cell counts are shown for the ambiguous scenarios in which all individuals are heterozygous: cell 9 for case-parent trios and cell 4 for case-mother duos and case-father duos. The estimated number of trios and duos in which the risk allele is inherited from the father is given as cells 9a and 4a, respectively, and the estimated number in which the risk allele is inherited from the mother is given as cells 9b and 4b, respectively. Cell counts estimated from duos need not be integers given that they incorporate the adjustment described in Appendix B. The odds ratios and p values given by EMIM, with and without haplotype estimation, are shown. Chromosomes 12 and 13 were tested for paternally and maternally inherited imprinting effects, respectively.

## Appendix A

### Derivation of Likelihood

Here, we derive the likelihood for the multinomial model when estimated haplotypes are used to estimate the parent of origin of alleles. The data considered consists of case-parent trios and control-parent trios and the methodology extends to other sub-pedigrees as previously described.^20,21^ We consider data observed at one SNP given that some, possibly all, of the data have been phased and parent-of-origin deduced. This gives an expression for the likelihood as follows:

$$L\left(\theta ;D\right)=\prod_{i}P\left(g_{m_{i}},g_{f_{i}},g_{c_{i}},{\text{Ψ}}_{i}|\theta ,{\text{dis}}_{i}\right),$$

where *D* is the data (consisting of genotype data $g_{m_{i}}$, $g_{f_{i}}$, and $g_{c_{i}}$ for the mother, father, and child, respectively, in the *i*^th^ trio and the event that the trio has been phased, Ψ_*i*_ = 1, or not, Ψ_*i*_ = 0), conditional on the child’s disease status dis_*i*_. The full set of multinomial model parameters is given by θ = (*R*_1_, *R*_2_, *S*_1_, *S*_2_, *I*_*m*_, *I*_*p*_, *γ*_11_, *γ*_12_, *γ*_21_, *γ*_22_, *μ*_1_, *μ*_2_, *μ*_3_, *μ*_4_, *μ*_5_, *μ*_6_) as defined by Ainsworth et al.^20^ We have

$$L\left(\theta ;D\right)=\prod_{i\in \text{diseased}}P\left(g_{m_{i}},g_{f_{i}},g_{c_{i}},{\text{Ψ}}_{i}|\theta ,{\text{dis}}_{i}=1\right)\prod_{i\in \text{not}\;\text{diseased}}P\left(g_{m_{i}},g_{f_{i}},g_{c_{i}},{\text{Ψ}}_{i}|\theta ,{\text{dis}}_{i}=0\right),$$

where “diseased” or “not diseased” relate to the sets of case subjects (dis_*i*_ = 1) or control subjects (dis_*i*_ = 0), respectively.

The first term of this product where the child is diseased gives

$$\begin{array}{l} \prod_{i\in \text{diseased}}P\left(g_{m_{i}},g_{f_{i}},g_{c_{i}},{\text{Ψ}}_{i}|\theta ,{\text{dis}}_{i}=1\right)=\\ \prod_{i\in \text{diseased},\text{ phased}}P\left(g_{m_{i}},g_{f_{i}},g_{c_{i}}|\theta ,{\text{dis}}_{i}=1,{\text{Ψ}}_{i}=1\right)P\left({\text{Ψ}}_{i}=1|\theta ,{\text{dis}}_{i}=1\right)\\ \times \prod_{i\in \text{diseased},\text{ not phased}}P\left(g_{m_{i}},g_{f_{i}},g_{c_{i}}|\theta ,{\text{dis}}_{i}=1,{\text{Ψ}}_{i}=0\right)P\left({\text{Ψ}}_{i}=0|\theta ,{\text{dis}}_{i}=1\right). \end{array}$$

Now, define *n*_*j*_ and $n_{j}^{\prime }$ as the number of case-parent trios with genotype data for cell *j*∈{1, …, 8, 9a, 9b, 10, …, 15} for not phased and phased data, respectively, where the 16 cells are defined as by Ainsworth et al.^20^ and $N_{j}=n_{j}+n_{j}^{\prime }$ as the total case-parent trios with diseased children for cell *j*. For phased data, $n_{9a}^{\text{′}}$ and $n_{9b}^{\prime }$ are calculated from the estimated haplotypes, and we can define cell 9 as the total of cells 9a and 9b so that $n_{9}^{\prime }=n_{9a}^{\prime }+n_{9b}^{\prime }$. For unphased data, cells 9a and 9b are not observed or estimated, so we again define cell 9 as the total of cells 9a and 9b, which is observed, so that *n*_9_ = *n*_9*a*_ + *n*_9*b*_.

Define *r* as the probability of a case-parent trio being phased, assuming independence with the genotypes. This is given by the proportion of trios that have been phased in the dataset. Referring to column 9 of Table 2 in Ainsworth et al,^20^ we then get

$$\prod_{i\in \text{diseased},\;\text{phased}}P\left(g_{m_{i}},g_{f_{i}},g_{c_{i}}|\theta ,{\text{dis}}_{i}=1,{\text{Ψ}}_{i}=1\right)P\left({\text{Ψ}}_{i}=1|{\text{dis}}_{i}=1\right)={\left[rR_{2}S_{2}I_{m}I_{p}\gamma_{22}\mu_{1}\right]}^{n_{1}^{\prime }}\times \ldots \times {\left[rR_{2}S_{1}I_{m}I_{p}\gamma_{12}\mu_{4}\right]}^{n_{8}^{\prime }}\times {\left[rR_{1}S_{1}I_{p}\gamma_{11}\mu_{4}\right]}^{n_{9a}^{\prime }}\times {\left[rR_{1}S_{1}I_{m}\gamma_{11}\mu_{4}\right]}^{n_{9b}^{\prime }}\times {\left[rS_{1}\mu_{4}\right]}^{n_{10}^{\prime }}\times {\left[r\mu_{6}\right]}^{n_{15}^{\prime }}.$$

When the data are not phased, we have *n*_9_ = *n*_9*a*_ + *n*_9*b*_. This then gives

$$\prod_{i\in \text{diseased},\;\text{not}\;\text{phased}}P\left(g_{m_{i}},g_{f_{i}},g_{c_{i}}|\theta ,{\text{dis}}_{i}=1,{\text{Ψ}}_{i}=0\right)P\left({\text{Ψ}}_{i}=0|{\text{dis}}_{i}=1\right)={\left[\left(1-r\right)R_{2}S_{2}I_{m}I_{p}\gamma_{22}\mu_{1}\right]}^{n_{1}}\times \ldots \times {\left[\left(1-r\right)R_{2}S_{1}I_{m}I_{p}\gamma_{12}\mu_{4}\right]}^{n_{8}}\times {\left[\left(1-r\right)R_{1}S_{1}\left(I_{p}+I_{m}\right)\gamma_{11}\mu_{4}\right]}^{n_{9}}\times {\left[\left(1-r\right)S_{1}\mu_{4}\right]}^{n_{10}}\times {\left[\left(1-r\right)\mu_{6}\right]}^{n_{15}}.$$

Therefore,

$$\prod_{i\in \text{diseased}}P\left(g_{m_{i}},g_{f_{i}},g_{c_{i}},{\text{Ψ}}_{i}|\theta ,{\text{dis}}_{i}=1\right)=r^{n_{1}^{\prime }}{\left(1-r\right)}^{n_{1}}\times \ldots \times r^{n_{8}^{\prime }}{\left(1-r\right)}^{n_{8}}\times r^{n_{10}^{\prime }}{\left(1-r\right)}^{n_{10}}\times \ldots \times r^{n_{15}^{\prime }}{\left(1-r\right)}^{n_{15}}\times {\left[R_{2}S_{2}I_{m}I_{p}\gamma_{22}\mu_{1}\right]}^{N_{1}}\times \ldots \times {\left[R_{2}S_{1}I_{m}I_{p}\gamma_{12}\mu_{4}\right]}^{N_{8}}\times {\left[rR_{1}S_{1}I_{p}\gamma_{11}\mu_{4}\right]}^{n_{9a}^{\prime }}\times {\left[rR_{1}S_{1}I_{m}\gamma_{11}\mu_{4}\right]}^{n_{9b}^{\prime }}\times {\left[\left(1-r\right)R_{1}S_{1}\left(I_{p}+I_{m}\right)\gamma_{11}\mu_{4}\right]}^{n_{9}}\times {\left[S_{1}\mu_{4}\right]}^{N_{10}}\times \ldots \times {\left[\mu_{6}\right]}^{N_{15}}.$$

Similarly, when the child is not diseased we use

$$\begin{array}{l} \prod_{i\in \text{not}\;\text{diseased}}P\left(g_{m_{i}},g_{f_{i}},g_{c_{i}},{\text{Ψ}}_{i}|\theta ,{\text{dis}}_{i}=0\right)=\\ \prod_{i\in \text{not}\;\text{diseased},\;\text{phased}}P\left(g_{m_{i}},g_{f_{i}},g_{c_{i}}|\theta ,{\text{dis}}_{i}=0,{\text{Ψ}}_{i}=1\right)P\left({\text{Ψ}}_{i}=1|\theta ,{\text{dis}}_{i}=0\right)\\ \times \prod_{i\in \text{not}\;\text{diseased},\;\text{not}\;\text{phased}}P\left(g_{m_{i}},g_{f_{i}},g_{c_{i}}|\theta ,{\text{dis}}_{i}=0,{\text{Ψ}}_{i}=0\right)P\left({\text{Ψ}}_{i}=1|\theta ,{\text{dis}}_{i}=0\right). \end{array}$$

Define *m*_*j*_ and $m_{j}^{\prime }$ as the number of control-parent trios with genotype data for each cell *j* for not phased and phased data, respectively, and $M_{j}=m_{j}+m_{j}^{\prime }$ as the total of control-parent trios with diseased children for cell *j*. Furthermore, define *r*′ as the probability of a control-parent trio being phased, assuming independence with the genotypes. As before, referring to Table 2 in Ainsworth et al,^20^ we obtain

$$\prod_{i\in \text{not}\;\text{diseased},\;\text{phased}}P\left(g_{m_{i}},g_{f_{i}},g_{c_{i}}|\theta ,{\text{dis}}_{i}=0,{\text{Ψ}}_{i}=1\right)P\left({\text{Ψ}}_{i}=1|{\text{dis}}_{i}=0\right)={\left[r^{\prime }\mu_{1}\right]}^{m_{1}^{\prime }}\times \ldots \times {\left[r^{\prime }\mu_{4}\right]}^{m_{8}^{\prime }}\times {\left[r^{\prime }\mu_{4}\right]}^{m_{9a}^{\prime }}\times {\left[r^{\prime }\mu_{4}\right]}^{m_{9b}^{\prime }}\times {\left[r^{\prime }\mu_{4}\right]}^{m_{10}^{\prime }}\times \ldots \times {\left[r^{\prime }\mu_{6}\right]}^{m_{15}^{\prime }}.$$

As with case-parent trios, we define cell 9 as the total of cells 9a and 9b, which is observed, so that *m*_9_ = *m*_9*a*_ + *m*_9*b*_. This then gives

$$\prod_{i\in \text{not}\;\text{diseased},\;\text{not}\;\text{phased}}P\left(g_{m_{i}},g_{f_{i}},g_{c_{i}}|\theta ,{\text{dis}}_{i}=0,{\text{Ψ}}_{i}=0\right)P\left({\text{Ψ}}_{i}=0|{\text{dis}}_{i}=0\right)={\left[\left(1-r^{\prime }\right)\mu_{1}\right]}^{m_{1}}\times \ldots \times {\left[\left(1-r^{\prime }\right)\mu_{4}\right]}^{m_{8}}\times {\left[\left(1-r^{\prime }\right)\mu_{4}\right]}^{m_{9}}\times {\left[\left(1-r^{\prime }\right)\mu_{4}\right]}^{m_{10}}\times \ldots \times {\left[\left(1-r^{\prime }\right)\mu_{6}\right]}^{m_{15}}.$$

Therefore,

$$\prod_{i\in \text{not diseased}}P\left(g_{m_{i}},g_{f_{i}},g_{c_{i}}|\theta ,{\text{dis}}_{i}=0\right)={\left(r^{\prime }\right)}^{m_{1}^{\prime }}{\left(1-r^{\prime }\right)}^{m_{1}}\times \ldots \times {\left(r^{\prime }\right)}^{m_{15}^{\prime }}{\left(1-r^{\prime }\right)}^{m_{15}}\times {\left[\mu_{1}\right]}^{M_{1}}\times \ldots \times {\left[\mu_{4}\right]}^{M_{8}}\times {\left[\mu_{4}\right]}^{M_{9}}\times {\left[\mu_{4}\right]}^{M_{10}}\times \ldots \times {\left[\mu_{6}\right]}^{M_{15}}.$$

Therefore, the likelihood of the data is

$$L\left(\theta ;D\right)=r^{n_{1}^{\prime }}{\left(1-r\right)}^{n_{1}}\times \ldots \times r^{n_{8}^{\prime }}{\left(1-r\right)}^{n_{8}}\times r^{n_{10}^{\prime }}{\left(1-r\right)}^{n_{10}}\times \ldots \times r^{n_{15}^{\prime }}{\left(1-r\right)}^{n_{15}}\times {\left(r^{\prime }\right)}^{m_{1}^{\prime }}{\left(1-r^{\prime }\right)}^{m_{1}}\times \ldots \times {\left(r^{\prime }\right)}^{m_{15}^{\prime }}{\left(1-r^{\prime }\right)}^{m_{15}}\times {\left[R_{2}S_{2}I_{m}I_{p}\gamma_{22}\mu_{1}\right]}^{N_{1}}\times {\left[R_{2}S_{2}I_{m}I_{p}\gamma_{22}\mu_{2}\right]}^{N_{2}}\times {\left[R_{1}S_{2}I_{m}\gamma_{21}\mu_{2}\right]}^{N_{3}}\times {\left[R_{2}S_{1}I_{m}I_{p}\gamma_{12}\mu_{2}\right]}^{N_{4}}\times {\left[R_{1}S_{1}I_{p}\gamma_{11}\mu_{2}\right]}^{N_{5}}\times {\left[R_{1}S_{2}I_{m}\gamma_{21}\mu_{3}\right]}^{N_{6}}\times {\left[R_{1}I_{p}\mu_{3}\right]}^{N_{7}}\times {\left[R_{2}S_{1}I_{m}I_{p}\gamma_{12}\mu_{4}\right]}^{N_{8}}\times {\left[rR_{1}S_{1}I_{p}\gamma_{11}\mu_{4}\right]}^{n_{9a}^{\prime }}\times {\left[rR_{1}S_{1}I_{m}\gamma_{11}\mu_{4}\right]}^{n_{9b}^{\prime }}\times {\left[\left(1-r\right)R_{1}S_{1}\left(I_{p}+I_{m}\right)\gamma_{11}\mu_{4}\right]}^{n_{9}}\times {\left[S_{1}\mu_{4}\right]}^{N_{10}}\times {\left[R_{1}S_{1}I_{m}\gamma_{11}\mu_{5}\right]}^{N_{11}}\times {\left[S_{1}\mu_{5}\right]}^{N_{12}}\times {\left[R_{1}I_{p}\mu_{5}\right]}^{N_{13}}\times {\left[\mu_{5}\right]}^{N_{14}}\times {\left[\mu_{6}\right]}^{N_{15}}\times {\left[\mu_{1}\right]}^{M_{1}}\times {\left[\mu_{2}\right]}^{M_{2}}\times {\left[\mu_{2}\right]}^{M_{3}}\times {\left[\mu_{2}\right]}^{M_{4}}\times {\left[\mu_{2}\right]}^{M_{5}}\times {\left[\mu_{3}\right]}^{M_{6}}\times {\left[\mu_{3}\right]}^{M_{7}}\times {\left[\mu_{4}\right]}^{M_{8}}\times {\left[\mu_{4}\right]}^{M_{9}}\times {\left[\mu_{4}\right]}^{M_{10}}\times {\left[\mu_{5}\right]}^{M_{11}}\times {\left[\mu_{5}\right]}^{M_{12}}\times {\left[\mu_{5}\right]}^{M_{13}}\times {\left[\mu_{5}\right]}^{M_{14}}\times {\left[\mu_{6}\right]}^{M_{15}}.$$

The log likelihood is therefore given by

$$l\left(\theta ;D\right)=\text{log}\left(L\left(\theta ;D\right)\right)=n_{1}^{\prime }\text{log}\left(r\right)+n_{1}\text{log}\left(1-r\right)+\ldots +n_{8}^{\prime }\text{log}\left(r\right)+n_{8}\text{log}\left(1-r\right)+n_{10}^{\prime }\text{log}\left(r\right)+n_{10}\text{log}\left(1-r\right)+\ldots +n_{15}^{\prime }\text{log}\left(r\right)+n_{15}\text{log}\left(1-r\right)+m_{1}^{\prime }\text{log}\left(r^{\prime }\right)+m_{1}\text{log}\left(1-r^{\prime }\right)+\ldots +m_{15}^{\prime }\text{log}\left(r^{\prime }\right)+m_{15}\text{log}\left(1-r^{\prime }\right)+N_{1}\text{log}\left(R_{2}S_{2}I_{m}I_{p}\gamma_{22}\mu_{1}\right)+N_{2}\text{log}\left(R_{2}S_{2}I_{m}I_{p}\gamma_{22}\mu_{2}\right)+N_{3}\text{log}\left(R_{1}S_{2}I_{m}\gamma_{21}\mu_{2}\right)+N_{4}\text{log}\left(R_{2}S_{1}I_{m}I_{p}\gamma_{12}\mu_{2}\right)+N_{5}\text{log}\left(R_{1}S_{1}I_{p}\gamma_{11}\mu_{2}\right)+N_{6}\text{log}\left(R_{1}S_{2}I_{m}\gamma_{21}\mu_{3}\right)+N_{7}\text{log}\left(R_{1}I_{p}\mu_{3}\right)+N_{8}\text{log}\left(R_{2}S_{1}I_{m}I_{p}\gamma_{12}\mu_{4}\right)+n_{9a}^{\prime }\text{log}\left(r\right)+n_{9b}^{\prime }\text{log}\left(r\right)+n_{9}\text{log}\left(1-r\right)+n_{9a}^{\prime }\text{log}\left(R_{1}S_{1}I_{p}\gamma_{11}\mu_{4}\right)+n_{9b}^{\prime }\text{log}\left(R_{1}S_{1}I_{m}\gamma_{11}\mu_{4}\right)+n_{9}\text{log}\left(R_{1}S_{1}\left(I_{p}+I_{m}\right)\gamma_{11}\mu_{4}\right)+N_{10}\text{log}\left(S_{1}\mu_{4}\right)+N_{11}\text{log}\left(R_{1}S_{1}I_{m}\gamma_{11}\mu_{5}\right)+N_{12}\text{log}\left(S_{1}\mu_{5}\right)+N_{13}\text{log}\left(R_{1}I_{p}\mu_{5}\right)+N_{14}\text{log}\left(\mu_{5}\right)+N_{15}\text{log}\left(\mu_{6}\right)+M_{1}\text{log}\left(\mu_{1}\right)+M_{2}\text{log}\left(\mu_{2}\right)+M_{3}\text{log}\left(\mu_{2}\right)+M_{4}\text{log}\left(\mu_{2}\right)+M_{5}\text{log}\left(\mu_{2}\right)+M_{6}\text{log}\left(\mu_{3}\right)+M_{7}\text{log}\left(\mu_{3}\right)+M_{8}\text{log}\left(\mu_{4}\right)+M_{9}\text{log}\left(\mu_{4}\right)+M_{10}\text{log}\left(\mu_{4}\right)+M_{11}\text{log}\left(\mu_{5}\right)+M_{12}\text{log}\left(\mu_{5}\right)+M_{13}\text{log}\left(\mu_{5}\right)+M_{14}\text{log}\left(\mu_{5}\right)+M_{15}\text{log}\left(\mu_{6}\right).$$

We note that, for fixed *r* and *r*′, the likehood does not depend on the values of *r* and *r*′, so these terms can be dropped. For convenience, in our software implementation, we retain *r* and *r*′ in the likelihood calculation, but we find the results are invariant to the choice of *r* and *r*′. This is equivalent to considering the overall likelihood as the product of the conditional likelihoods *P*(*g*_*m*_*i*__, *g*_*f*_*i*__, *g*_*c*_*i*__ | θ, dis_*i*_, Ψ_*i*_ = 0) and *P*(*g*_*m*_*i*__, *g*_*f*_*i*__, *g*_*c*_*i*__ | θ, dis_*i*_, Ψ_*i*_ = 1) for the unphased (15 cell) and phased (16 cell) tables, respectively.

For other types of sub-pedigrees, including case-mother duos and case-father duos, the calculation proceeds similarly.

## Appendix B

### Adjustment of Genotype Counts for Duos

Let *N* be the number of case-mother duos and *M* the number of SNPs to be analyzed. (Genotype counts for case-father duos are adjusted in the same manner, but we only consider case-mother duos here for simplicity.) Let *X* = {*x*_1_,…,*x*_*M*_} be the sequence of observed SNP case-mother duo data such that each *x*_*i*_ is a vector of cell counts {*x*_*ij*_} for SNP *i* for case-mother duo cells *j* = 1, 2, 3, 4a, 4b, 5, 6, and 7. Let *p*_*i*_ be the minor allele frequency of SNP *i*, then, assuming Hardy-Weinberg equilibrium and random mating, *x*_*i*_ is given by a multinomial distribution with $\sum_{j}x_{ij}=N$ and eight probabilities θ_*ij*_, with probabilities for cells 4a and 4b given by $\theta_{i\left(4a\right)}=p_{i}^{2}\left(1-p_{i}\right)$ and $\theta_{i\left(4b\right)}=p_{i}{\left(1-p_{i}\right)}^{2}$, respectively. Let ${\text{Σ}}_{i}$ be the covariance matrix where the variances for cells 4a and 4b are given by ${\text{Σ}}_{i\left(4a,4a\right)}=N\theta_{i\left(4a\right)}\left(1-\theta_{i\left(4a\right)}\right)$ and ${\text{Σ}}_{i\left(4b,4b\right)}=N\theta_{i\left(4b\right)}\left(1-\theta_{i\left(4b\right)}\right)$, respectively, with covariance ${\text{Σ}}_{i\left(4a,4b\right)}=-N\theta_{i\left(4a\right)}\theta_{i\left(4b\right)}$.

Approximating the multinomial distribution with the multivariate normal distribution gives the following likelihood function:

$$L\left(X\right)=\prod_{i=1}^{M}\frac{1}{\sqrt{{\left(2\pi \right)}^{2}\left|{\text{Σ}}_{i}\right|}}\text{exp}\left(-\frac{1}{2}{\left(x_{i}-\mu_{i}\right)}^{T}{\text{Σ}}_{i}^{-1}\left(x_{i}-\mu_{i}\right)\right),$$

where *μ*_*i*_ = *N*θ_*i*_ are the expected cell counts for SNP *i*. To correct the bias, we add a correction vector, *F*(*p*_*i*_), to the observed data, with *F*(*p*_*i*_) given by

$$F\left(p_{i}\right)={\left(0,0,0,-Nf\left(p_{i}\right),Nf\left(p_{i}\right),0,0,0\right)}^{T},$$

where *f*(*p*) = β_0_ + β_1_*p* + β_2_*p*^2^ + β_3_*p*^3^, so that the cell counts 4a and 4b are adjusted while maintaining the sum $\sum_{j}x_{ij}=N$. We choose β = {β_0_,β_1_,β_2_,β_3_} to maximize the likelihood:

$$L\left(X|\beta \right)=\prod_{i=1}^{M}\frac{1}{\sqrt{{\left(2\pi \right)}^{2}\left|{\text{Σ}}_{i}\right|}}\times \text{exp}\left(-\frac{1}{2}{\left(x_{i}+F\left(p_{i}\right)-\mu_{i}\right)}^{T}{\text{Σ}}_{i}^{-1}\left(x_{i}+F\left(p_{i}\right)-\mu_{i}\right)\right).$$

It was found that higher order polynomials than cubic resulted in over fitting, whereas lower order polynomials were insufficient to model the bias over the different minor allele frequencies. Taking the negative logarithm and removing all constants that do not depend on β results in the following negative log likelihood:

$$l\left(X|\beta \right)=\overset{M}{\sum_{i=1}}\frac{1}{2\left({\text{Σ}}_{i\left(4a,4a\right)}{\text{Σ}}_{i\left(4b,4b\right)}-{\text{Σ}}_{i\left(4a,4b\right)}\right)}\times [{\left(x_{i\left(4a\right)}-Nf\left(p_{i}\right)-\mu_{i\left(4a\right)}\right)}^{2}{\text{Σ}}_{i\left(4b,4b\right)}-2\left(x_{i\left(4a\right)}-Nf\left(p_{i}\right)-\mu_{i\left(4a\right)}\right)\left(x_{i\left(4b\right)}-Nf\left(p_{i}\right)-\mu_{i\left(4b\right)}\right){\text{Σ}}_{i\left(4a,4b\right)}+{\left(x_{i\left(4b\right)}+Nf\left(p_{i}\right)-\mu_{i\left(4b\right)}\right)}^{2}{\text{Σ}}_{i\left(4a,4a\right)}],$$

where *x*_*i*(4*a*)_ and *x*_*i*(4*b*)_ are the number of case-mother duos (with expected values $\mu_{i\left(4a\right)}=N\theta_{i\left(4a\right)}=Np_{i}^{2}\left(1-p_{i}\right)$ and $\mu_{i\left(4b\right)}=N\theta_{i\left(4b\right)}=Np_{i}{\left(1-p_{i}\right)}^{2}$) for cells 4a and 4b, respectively. Newton’s Method is used to fit β in PREMIM by minimizing the negative log-likelihood with (fixed) estimated minor allele frequencies, *p*_*i*_, for each SNP *i*. The new adjusted cell counts for cells 4a and 4b are then given by *x*_*i*(4*a*)_ − *Nf*(*p*_*i*_) and *x*_*i*(4*b*)_ + *Nf*(*p*_*i*_), respectively.

### Example Illustrating the Adjustment of Genotype Counts for Duos

A demonstration of adjusting the duo counts is shown in Figure S1. Data were simulated under the null hypothesis for 1,500 case-mother duos with haplotypes of eight SNPs in length repeated 25 times, which gave rise to 200 SNPs with expected minor allele frequencies of 0.34, 0.45, 0.35, 0.25, 0.20, 0.16, 0.12, and 0.08, shown by the clustering of points in Figure S1 around these values. The top left plot shows, for each SNP, the SHAPEIT2 estimated number of minor alleles inherited from the father for the ambiguous scenario (cell 4a) plotted against the estimated minor allele frequency. The solid line shows the expected number of minor alleles inherited from the father under the null hypothesis, and it can be seen that the clusters of points tend to be not centered on this line but are too high for low minor allele frequencies and too low for high minor allele frequencies. The corresponding plots for the number of minor alleles inherited from the mother (cell 4b) shows a similar, but reversed, pattern. This can be seen more clearly with the estimated cell count minus the expected cell count (bottom left plots) where the points are not clustered around zero.

The dashed lines on the two top left plots in Figure S1 show the fitted curves used to adjust the genotype counts, *N*(*p*^2^(1 − *p*) + *f*(*p*)) and *N*(*p*(1 − *p*)^2^ − *f*(*p*)) for *p*∈[0, 0.5], for the number of minor alleles inherited from the father and mother, respectively. These two curves are fitted simultaneously, and if the estimates given by SHAPEIT2 are accurate, then these should be close to the expected curves, with the fitted parameters β being close to zero. In the example in Figure S1 this is not true (although the tails of the dashed lines can be ignored given that there is no data in these regions). The dashed lines on the two lower plots on the left show *Nf*(*p*) and −*Nf*(*p*), respectively, and show how the estimated cell counts will be adjusted.

The two top right plots in Figure S1 show that the adjusted cell counts are nearer the expected curves than the unadjusted cell counts. This is more clearly shown on the two lower right plots where the differences between the estimated and the expected (under the null hypothesis) cell counts are shown.

## Web Resources

The URLs for data presented herein are as follows:

OMIM, http://www.omim.org
PREMIM and EMIM, https://www.staff.ncl.ac.uk/richard.howey/emim/index.html
SHAPEIT2, https://mathgen.stats.ox.ac.uk/genetics_software/shapeit/shapeit.html#home

## Supplemental Data

Document S1. Figures S1–S6

Document S2. Article plus Supplemental Data

## References

[1] Lawson H.A., Cheverud J.M., Wolf J.B. (2013). Genomic imprinting and parent-of-origin effects on complex traits. Nat. Rev. Genet..

[2] Mott R., Yuan W., Kaisaki P., Gan X., Cleak J., Edwards A., Baud A., Flint J. (2014). The architecture of parent-of-origin effects in mice. Cell.

[3] Guilmatre A., Sharp A.J. (2012). Parent of origin effects. Clin. Genet..

[4] Wallace C., Smyth D.J., Maisuria-Armer M., Walker N.M., Todd J.A., Clayton D.G. (2010). The imprinted DLK1-MEG3 gene region on chromosome 14q32.2 alters susceptibility to type 1 diabetes. Nat. Genet..

[5] Kong A., Steinthorsdottir V., Masson G., Thorleifsson G., Sulem P., Besenbacher S., Jonasdottir A., Sigurdsson A., Kristinsson K.T., Jonasdottir A., DIAGRAM Consortium (2009). Parental origin of sequence variants associated with complex diseases. Nature.

[6] Nudel R., Simpson N.H., Baird G., O’Hare A., Conti-Ramsden G., Bolton P.F., Hennessy E.R., Ring S.M., Davey Smith G., Francks C., SLI Consortium (2014). Genome-wide association analyses of child genotype effects and parent-of-origin effects in specific language impairment. Genes Brain Behav..

[7] Esparza-Gordillo J., Matanovic A., Marenholz I., Bauerfeind A., Rohde K., Nemat K., Lee-Kirsch M.A., Nordenskjöld M., Winge M.C., Keil T. (2015). Maternal filaggrin mutations increase the risk of atopic dermatitis in children: an effect independent of mutation inheritance. PLoS Genet..

[8] Jamieson S.E., de Roubaix L.A., Cortina-Borja M., Tan H.K., Mui E.J., Cordell H.J., Kirisits M.J., Miller E.N., Peacock C.S., Hargrave A.C. (2008). Genetic and epigenetic factors at COL2A1 and ABCA4 influence clinical outcome in congenital toxoplasmosis. PLoS ONE.

[9] Kistner E.O., Infante-Rivard C., Weinberg C.R. (2006). A method for using incomplete triads to test maternally mediated genetic effects and parent-of-origin effects in relation to a quantitative trait. Am. J. Epidemiol..

[10] Wheeler E., Cordell H.J. (2007). Quantitative trait association in parent offspring trios: Extension of case/pseudocontrol method and comparison of prospective and retrospective approaches. Genet. Epidemiol..

[11] Belonogova N.M., Axenovich T.I., Aulchenko Y.S. (2010). A powerful genome-wide feasible approach to detect parent-of-origin effects in studies of quantitative traits. Eur. J. Hum. Genet..

[12] Hoggart C.J., Venturini G., Mangino M., Gomez F., Ascari G., Zhao J.H., Teumer A., Winkler T.W., Tšernikova N., Luan J., Generation Scotland Consortium, LifeLines Cohort study, GIANT Consortium (2014). Novel approach identifies SNPs in SLC2A10 and KCNK9 with evidence for parent-of-origin effect on body mass index. PLoS Genet..

[13] Connolly S., Heron E.A. (2015). Review of statistical methodologies for the detection of parent-of-origin effects in family trio genome-wide association data with binary disease traits. Brief. Bioinform..

[14] Purcell S., Neale B., Todd-Brown K., Thomas L., Ferreira M.A., Bender D., Maller J., Sklar P., de Bakker P.I., Daly M.J., Sham P.C. (2007). PLINK: a tool set for whole-genome association and population-based linkage analyses. Am. J. Hum. Genet..

[15] Spielman R.S., McGinnis R.E., Ewens W.J. (1993). Transmission test for linkage disequilibrium: the insulin gene region and insulin-dependent diabetes mellitus (IDDM). Am. J. Hum. Genet..

[16] Weinberg C.R. (1999). Methods for detection of parent-of-origin effects in genetic studies of case-parents triads. Am. J. Hum. Genet..

[17] Weinberg C.R., Wilcox A.J., Lie R.T. (1998). A log-linear approach to case-parent-triad data: assessing effects of disease genes that act either directly or through maternal effects and that may be subject to parental imprinting. Am. J. Hum. Genet..

[18] Zhou J.Y., Hu Y.Q., Fung W.K. (2007). A simple method for detection of imprinting effects based on case-parents trios. Heredity (Edinb).

[19] Cordell H.J., Barratt B.J., Clayton D.G. (2004). Case/pseudocontrol analysis in genetic association studies: A unified framework for detection of genotype and haplotype associations, gene-gene and gene-environment interactions, and parent-of-origin effects. Genet. Epidemiol..

[20] Ainsworth H.F., Unwin J., Jamison D.L., Cordell H.J. (2011). Investigation of maternal effects, maternal-fetal interactions and parent-of-origin effects (imprinting), using mothers and their offspring. Genet. Epidemiol..

[21] Howey R., Cordell H.J. (2012). PREMIM and EMIM: tools for estimation of maternal, imprinting and interaction effects using multinomial modelling. BMC Bioinformatics.

[22] Becker T., Baur M.P., Knapp M. (2006). Detection of parent-of-origin effects in nuclear families using haplotype analysis. Hum. Hered..

[23] Zhou J.Y., Lin S., Fung W.K., Hu Y.Q. (2009). Detection of parent-of-origin effects in complete and incomplete nuclear families with multiple affected children using multiple tightly linked markers. Hum. Hered..

[24] Gjessing H.K., Lie R.T. (2006). Case-parent triads: estimating single- and double-dose effects of fetal and maternal disease gene haplotypes. Ann. Hum. Genet..

[25] Shi M., Umbach D.M., Weinberg C.R. (2009). Using case-parent triads to estimate relative risks associated with a candidate haplotype. Ann. Hum. Genet..

[26] Zhang K., Sun F., Zhao H. (2005). HAPLORE: a program for haplotype reconstruction in general pedigrees without recombination. Bioinformatics.

[27] Dudbridge F. (2008). Likelihood-based association analysis for nuclear families and unrelated subjects with missing genotype data. Hum. Hered..

[28] Myers S., Bottolo L., Freeman C., McVean G., Donnelly P. (2005). A fine-scale map of recombination rates and hotspots across the human genome. Science.

[29] Frazer K.A., Ballinger D.G., Cox D.R., Hinds D.A., Stuve L.L., Gibbs R.A., Belmont J.W., Boudreau A., Hardenbol P., Leal S.M., International HapMap Consortium (2007). A second generation human haplotype map of over 3.1 million SNPs. Nature.

[30] Marchini J., Howie B. (2010). Genotype imputation for genome-wide association studies. Nat. Rev. Genet..

[31] Delaneau O., Marchini J., Zagury J.F. (2012). A linear complexity phasing method for thousands of genomes. Nat. Methods.

[32] Delaneau O., Zagury J.F., Marchini J. (2013). Improved whole-chromosome phasing for disease and population genetic studies. Nat. Methods.

[33] Leal S.M., Yan K., Müller-Myhsok B. (2005). SimPed: a simulation program to generate haplotype and genotype data for pedigree structures. Hum. Hered..

[34] Anderson C.A., Pettersson F.H., Clarke G.M., Cardon L.R., Morris A.P., Zondervan K.T. (2010). Data quality control in genetic case-control association studies. Nat. Protoc..

[35] Cordell H.J., Töpf A., Mamasoula C., Postma A.V., Bentham J., Zelenika D., Heath S., Blue G., Cosgrove C., Granados Riveron J. (2013). Genome-wide association study identifies loci on 12q24 and 13q32 associated with tetralogy of Fallot. Hum. Mol. Genet..

[36] Devlin B., Roeder K. (1999). Genomic control for association studies. Biometrics.

[37] Morison I.M., Ramsay J.P., Spencer H.G. (2005). A census of mammalian imprinting. Trends Genet..

[38] Glaser R.L., Ramsay J.P., Morison I.M. (2006). The imprinted gene and parent-of-origin effect database now includes parental origin of de novo mutations. Nucleic Acids Res..

[39] Kotzot D. (2001). Comparative analysis of isodisomic and heterodisomic segments in cases with maternal uniparental disomy 14 suggests more than one imprinted region. Clin. Genet..

[40] Sutton V.R., Shaffer L.G. (2000). Search for imprinted regions on chromosome 14: comparison of maternal and paternal UPD cases with cases of chromosome 14 deletion. Am. J. Med. Genet..

