# Supplementary material for: Increased Power for Detection of Parent-of-Origin Effects via the Use of Haplotype Estimation
Source: Am J Hum Genet. 2015 Aug 27;97(3):419–34. doi: 10.1016/j.ajhg.2015.07.016 (PMC4564992; doi:10.1016/j.ajhg.2015.07.016)
Supplement: Document S1. Figures S1–S6 [file mmc1.pdf]

The American Journal of Human Genetics
Supplemental Data

# Increased Power for Detection of Parent-of-Origin Effects via the Use of Haplotype Estimation

Richard Howey, Chrysovalanto Mamasoula, Ana Töpf, Ron Nudel,
Judith A. Goodship, Bernard D. Keavney, and Heather J. Cordell

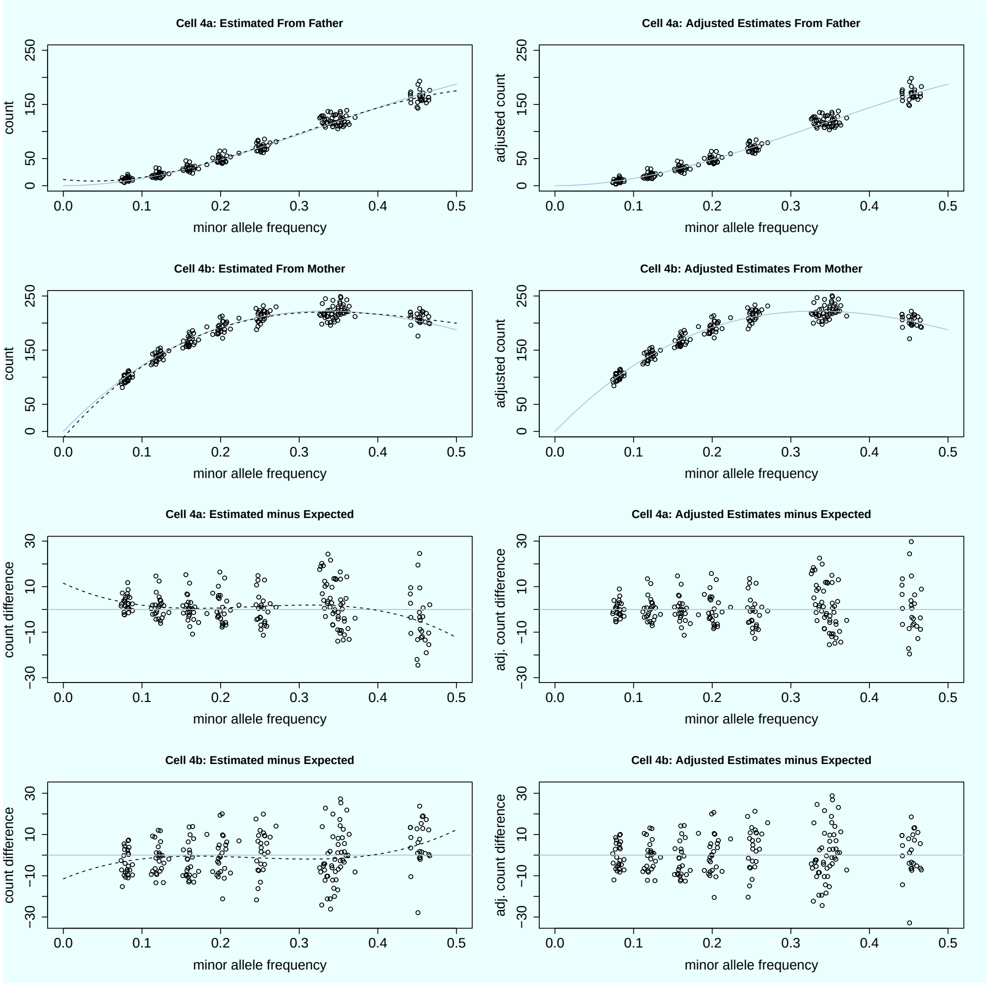


**Figure S1:** Plots of the estimated parent-of-origin of case/mother duo data for the ambiguous scenario where the mother and child are heterozygous. Left column: SHAPEIT2 estimates. Right column: adjusted SHAPEIT2 estimates. Top: estimated cell counts for the number of risk alleles inherited from the father. Second row: estimated cell counts for the number of risk alleles inherited from the mother. Third row: estimated cell counts minus the expected cell counts for the number of risk alleles inherited from the father. Fourth row: estimated cell counts minus the expected cell counts for the number of risk alleles inherited from the mother. Data was simulated using 1500 case/mother duos with 200 SNPs arranged in haplotypes of length 8. The solid curves show the expected cell counts for different minor allele frequencies. The dashed lines show the fitted curves used to adjust the estimated cell counts. Expected cell counts are calculated assuming HWE and random mating under the null hypothesis that no parent-of-origin effects exist.

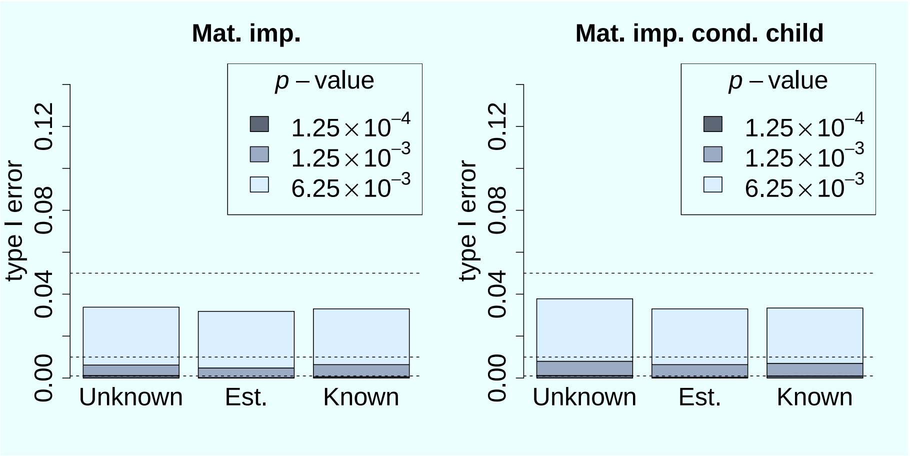


**Figure S2:** Bar plots of type I errors of EMIM (for $p$-value thresholds $1.25 \times 10^{-4}$, $1.25 \times 10^{-3}$ and $6.25 \times 10^{-3}$) using simulated case/parent trio data. Unknown: No haplotype estimation performed; Est.: parent-of-origin estimated using SHAPEIT2; Known: parent-of-origin assumed known. The left plot shows the type I error when testing for maternally inherited imprinting effects and the right plot shows the type I error when testing for maternally inherited imprinting effects conditional on child genotype effects.

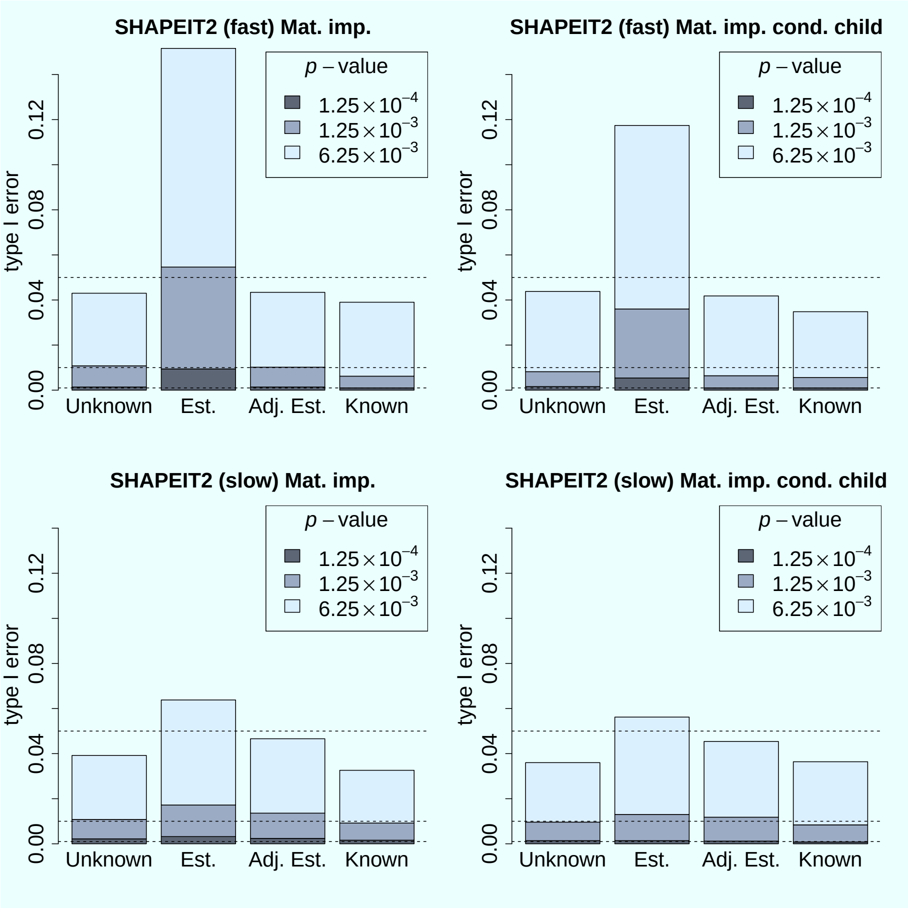


**Figure S3:** Bar plots of type I errors of EMIM (for $p$-value thresholds $1.25 \times 10^{-4}$, $1.25 \times 10^{-3}$ and $6.25 \times 10^{-3}$) using simulated case/mother duo data. Unknown: No haplotype estimation performed; Est.: parent-of-origin estimated using SHAPEIT2 with and without adjustment; Known: parent-of-origin assumed known. The top plots use SHAPEIT2 options designed to speed up the SHAPEIT2 analysis, while the bottom plots use the slower default options. The left plots show the type I error when testing for maternally inherited imprinting effects and the right plots show the type I error when testing for maternally inherited imprinting effects conditional on child genotype effects. Dashed lines show the expected family wise error rates assuming the 8 SNPs are independent.

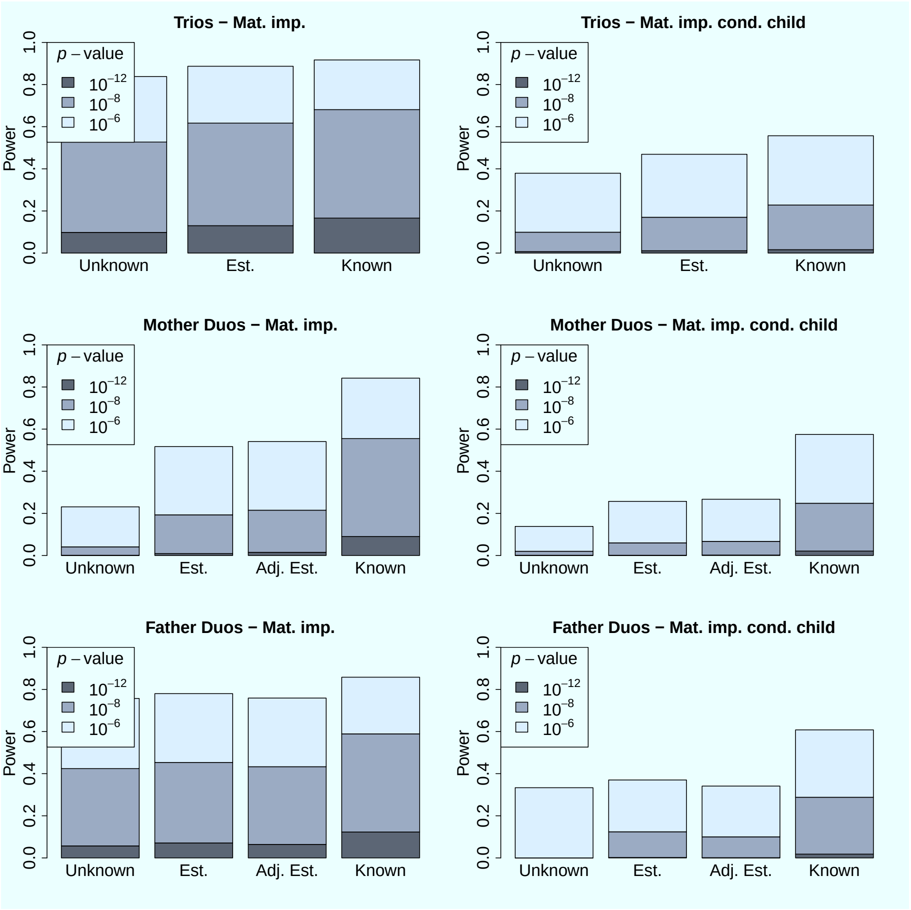


**Figure S4:** Bar plots of the powers of EMIM (for $p$-value thresholds $10^{-12}$, $10^{-10}$ and $10^{-6}$) using simulated case/parent trio, case/mother duo or case/father duo data, assuming a causal SNP with maternally inherited imprinting effect $I_m = 1.5$. Unknown: No haplotype estimation performed; Est.: parent-of-origin estimated using SHAPEIT2 with and without adjustment; Known: parent-of-origin assumed known. The left plots show the power to detect maternally inherited imprinting effects and the right plots show the power to detect maternally inherited imprinting effects conditional on child genotype effects.

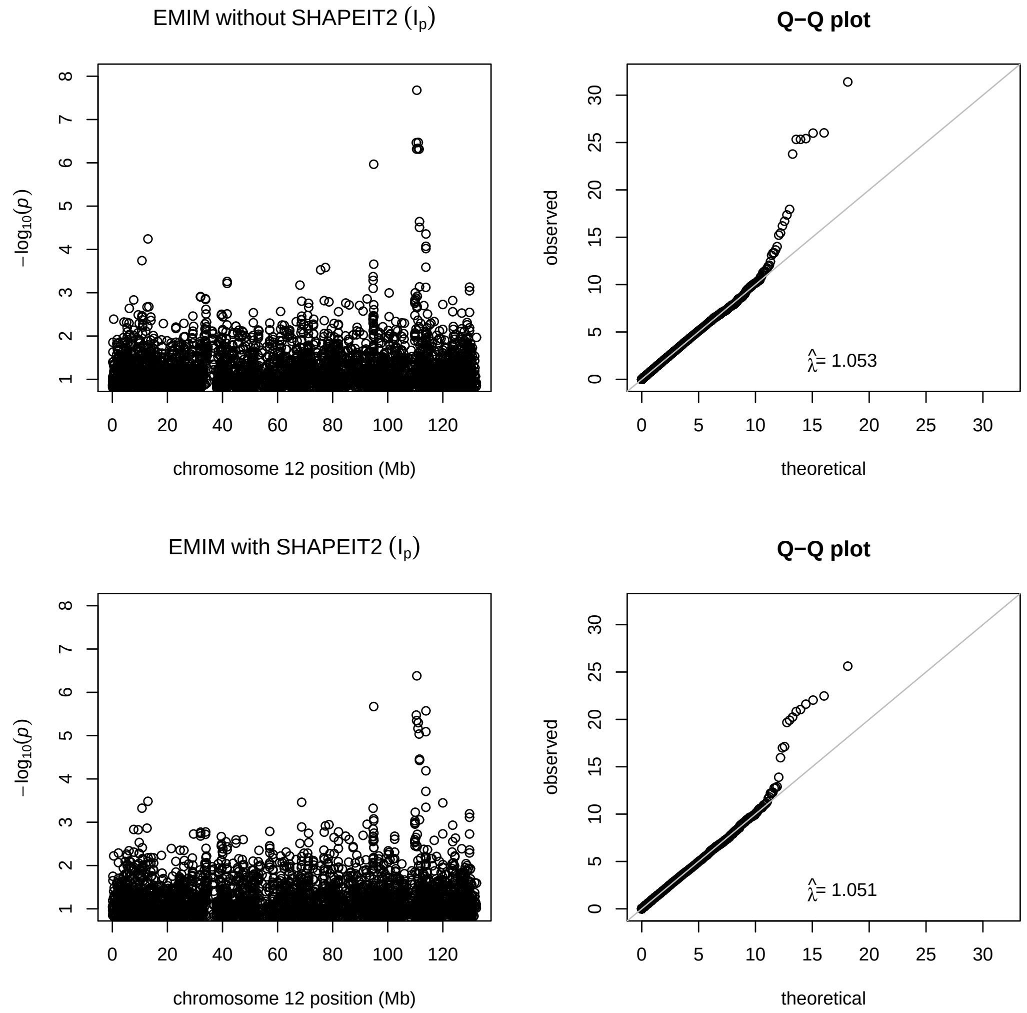


**Figure S5:** Manhattan plots of the $-\log_{10}$ $p$-values and Q-Q plots of the test statistics on chromosome 12, testing for paternally inherited imprinting in the tetralogy of Fallot data using EMIM alone (top plots) and EMIM with SHAPEIT2 (bottom plots). $\hat{\lambda}$ indicates the genomic control inflation factor.

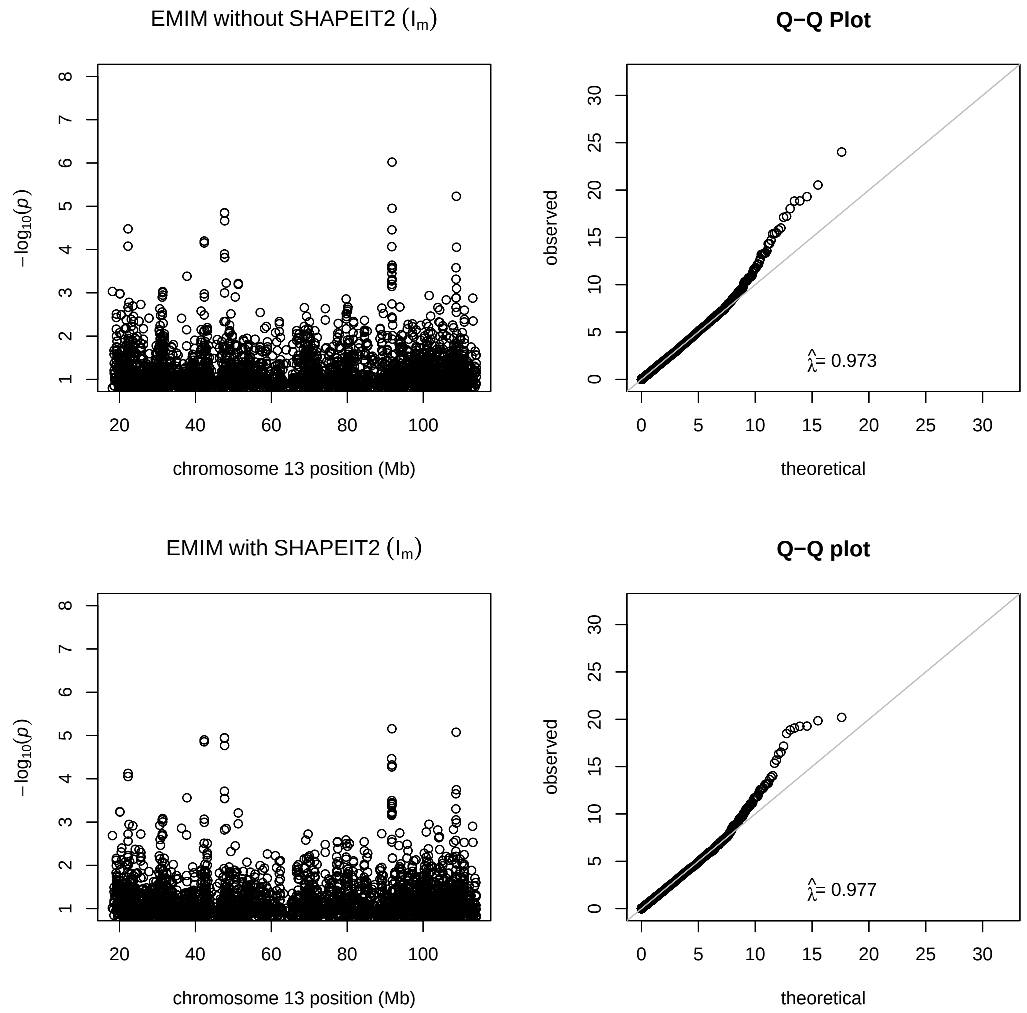


**Figure S6:** Manhattan plots of the $-\log_{10}$ $p$-values and Q-Q plots of the test statistics on chromosome 13, testing for maternally inherited imprinting in the tetralogy of Fallot data using EMIM alone (top plots) and EMIM with SHAPEIT2 (bottom plots). $\hat{\lambda}$ indicates the genomic control inflation factor.
